# Complex Network and QTAIM Analyses Reveal the Dominance of Structural over Electronic Modulation in Supercritical Water under Ion-Free and Ionic Conditions

**DOI:** 10.1021/acsomega.6c02186

**Published:** 2026-04-29

**Authors:** Artur G. Nogueira, Angélica Sousa da Mata, Teodorico C. Ramalho

**Affiliations:** † Department of Chemistry, 67739 Federal University of Lavras, Lavras, MG 37200-000, Brazil; ‡ Department of Physics, 67739 Federal University of Lavras, Lavras, MG 37200-000, Brazil; ¶ Centre for Basic and Applied Research, Faculty of Informatics and Management, University of Hradec Králové, Hradec Králové 500 03, Czech Republic

## Abstract

Supercritical fluids play a fundamental role in a wide range of scientific, industrial, environmental, and engineering processes due to the presence of inhomogeneities in their microscopic structure, which characterize a continuous transition between two structurally distinct regimes: a low-density gas-like state and a high-density liquid-like state. However, the relationship between thermodynamic conditions and the microstructural organization of water under supercritical conditions is still under investigation. In this work, we employ criteria based on energetic principles to characterize intermolecular interactions combined with network topological properties to describe the microstructural organization of water across variations in temperature and pressure in the supercritical regime. Analysis based on the quantum theory of atoms in molecules (QTAIM) indicates that intermolecular interactions between water molecules are predominantly noncovalent in nature, although partially covalent contributions may emerge within more extensively connected components under specific conditions. Statistical analyses further demonstrate that thermodynamically induced variations manifest primarily at a collective and topological level of the molecular network without significant changes in the local electronic nature of intermolecular interactions.

## Graphical abstract

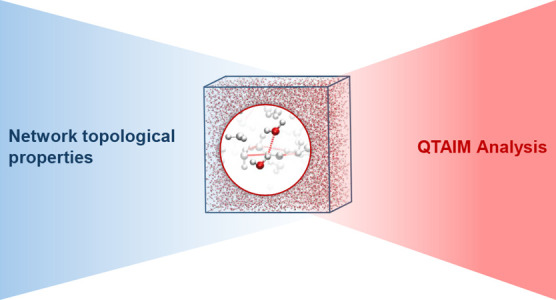

## Introduction

Water is a fundamental chemical substance, simple, and essential for sustaining life on our planet. It acts as a solvent in metabolic and industrial processes for the synthesis of compounds. However, the significance of water goes beyond its chemical formula, encompassing cultural, social, economic, and scientific aspects.^1^ Under normal conditions, as we know in our daily lives, water acts as a polar solvent with a low solubility in organic compounds. However, under supercritical conditions (temperature greater than 647.096 K and pressure greater than 220.640 bar), significant changes occur in its properties: the diffusion coefficient and oxidation efficiency increase substantially, and the dielectric constant and polarity are drastically reduced, the surface tension approaches zero, and the hydrogen bonding system is practically broken down.^2–5^

Although supercritical fluids (SCFs) are widely used in various industrial applications due to their unique properties, the relationship between thermodynamic conditions and microstructural behavior remains poorly understood.^6^ In particular, the emergence of transient microstructures, such as molecular clusters and density fluctuations,^7^ raises questions about the fundamental mechanisms governing these dynamics in supercritical regimes. This knowledge gap hinders the prediction and optimization of SCF performance in industrial systems, where stability, heat transfer efficiency,^8,9^ and system homogeneity are crucial.

An innovative approach adopted in this study is the use of complex network science to characterize clusters of water molecules under supercritical conditions, integrating principles of classical physics to describe connectivity and molecular dynamics. Unlike conventional methodologies based solely on radial distribution functions or statistical analyses of intermolecular interactions, the network-based approach captures the collective organization of molecules beyond local correlations. Specifically, it enables the identification of mesoscopic structural features, including the topology of molecular clusters, connectivity patterns, and the presence of transient, spatially extended structures arising from cooperative interactions. Additionally, network metrics provide quantitative measures of structural heterogeneity under varying thermodynamic conditions. This framework therefore reveals how local intermolecular interactions propagate through the system to generate emergent structural organization,^10^ offering insights that are not directly accessible through conventional statistical or correlation-based methods.^11,12^

In addition, this study discusses the nature of intermolecular interactions of water molecules under supercritical conditions using quantum theory of atoms in molecules (QTAIM) fundamentals and introduces a parametrized model that employs an adjustable cylindrical radius to minimize interference from third molecules when assessing the interaction potential between molecular pairs. This approach reduces noise arising from random fluctuations in the medium, enabling a more accurate estimation of the stability of pairwise interactions in a supercritical environment. By identifying which molecular pairs are most likely to form stable clusters, this study offers a new perspective on the fundamental mechanisms governing water organization in supercritical fluids, contributing to the development of more robust predictive models. The primary objective of this work is to investigate how thermodynamic conditions affect the organization of the intermolecular interaction network in water and the nature of these interactions. Additionally, this study aimed to determine whether electronic factors or topological parameters govern the thermodynamic properties of supercritical water.

## Methods

### Molecular Dynamics Simulations

To understand the structural dynamics of supercritical water, the molecular dynamics simulation was performed using GROningen MAchine for Chemical Simulations (GROMACS) version 5.1.2.^13^ A total of 20 simulations were initially performed under reduced temperature conditions Tr = 1.05, 1.10, 1.15, and 1.20 (eq 1) and reduced pressure conditions Pr = 2.50, 2.75, 3.00, 3.25, and 3.50 (eq 2). Each simulation comprised 8544 water molecules described by the TIP4*P*/2005 model^14^ with the OPLS-AA force field,^15^ in boxes whose dimensions were adjusted according to the experimental density corresponding to each condition.^16^ The reduced variables are defined as

$$\mathrm{Tr}=\frac{T}{647.096\;K}$$ 1

$$\mathrm{Pr}=\frac{P}{220.64\;\mathrm{bar}}$$ 2

Two energy minimization steps were performed using the Verlet cutoff scheme, consisting of 1000 and 50,000 steps, respectively. The difference between them was the application of the LINCS constraint algorithm in only the first minimization step. After the energy minimization steps, the system underwent two equilibration phases with a time step of 2.0 fs: (i) 1.0 ns under the NVT (canonical) ensemble using a velocity rescaling thermostat,^17^ followed by (ii) 1.0 ns under the NPT (isothermal–isobaric) ensemble with the same thermostat and the Berendsen barostat.^18^ For all simulations, periodic boundary conditions were applied in the three spatial directions, and the LINCS algorithm^19^ was employed to constrain hydrogen bonds. Long-range electrostatic interactions were treated using the Particle Mesh Ewald (PME) method.^20^ A cutoff distance of 1.0 nm was applied for short-range nonbonding interactions, including electrostatic and van der Waals contributions, applying long-range dispersion corrections for the Energy and Pressure.

After the equilibration steps, 50 ns production simulations were performed with a time step of 2.0 fs, using the Nose–Hoover thermostat^21,22^ and the Parrinello–Rahman barostat^23,24^ while keeping the remaining settings consistent with the previous NPT stage. Upon completion of the production stage, the average temperature, pressure, and density were calculated during the simulation and compared with the experimental data available in the NIST database.^16^ For subsequent analyses, only the simulations with a density deviation lower than 5.00% were considered. Based on the thermodynamic conditions with the lowest error, additional simulations were carried out under these conditions, but with the addition of Cl^–^ ions at a concentration of 0.20 M.^25^ This concentration was chosen because it falls within the dilute regime, where ion–ion correlations remain limited, allowing the system to capture local ionic hydration effects without introducing strong collective electrostatic interactions. This choice is consistent with previous molecular studies on halide hydration.^25–27^ The inclusion of Cl^–^ ions is not intended to represent a realistic electrolyte solution but rather to probe the robustness of the observed network properties under localized electrostatic perturbations.

Furthermore, a simulation was performed under the reduced conditions Tr = 1.04 and Pr = 2.27 in order to validate the model through comparisons between the radial distribution functions (RDFs) *g*_OO_, *g*_OH_, and *g*_HH_ obtained from the simulation and the corresponding experimental RDFs^28^ under the same temperature and pressure conditions. To quantify the degree of agreement between the simulated and experimental RDFs, *R* ^2^, root-mean-squared error (*RMSE*)*,* and mean absolute error (*MAE*) metrics were employed.

Once the production stage was completed, a dataframe was constructed in which each row corresponds to a frame of the molecular dynamics simulation, with a time step of 0.1 ns, and each column represents a spatial coordinate (*x*, *y*, *z*) of each atomic site of the TIP4*P*/2005 water model (H_a_, O, M, and H_b_) for each of the 8544 water molecules present in the simulation, totaling 102,528 columns. From this dataframe, a principal component analysis (PCA) was performed using Python code with the NumPy^29^ and scikit-learn^30,31^ libraries, and the three frames closest to the mean of the distribution in the new sample space were selected as representative of the respective simulations.

### Networks of Intermolecular Interactions

For the construction of the topological interaction networks, each water molecule from the representative frames was considered as a node, while the connections between these nodes represent intermolecular interactions. These connections were established according to the criteria defined by eq 3. When the interaction condition between two molecules is satisfied, the water molecule *j* is added to a set of molecules *C*_*m*_^(*t*)^, provided that it exhibits a significant energetic connection with a molecule *i* already belonging to the same set *C*_*m*_^(*t*)^:

$$C_{m}^{\left(t\right)}:=\{j|(\exists i\in C_{m}^{\left(t\right)})[V_{\mathrm{Coulomb}}^{ij}+V_{\mathrm{LJ}}^{ij}+E_{\mathrm{Kin}}^{ij}+\lambda \cdot 4E_{\mathrm{Kin}}^{H}<0]\}$$ 3

These calculations were performed with Python algorithms specifically developed for the methodology adopted in this study. For the calculation of the Lennard-Jones energy, *V*_LJ_^*ij*^, only the potential corresponding to the interaction between the oxygen atoms *O*_*i*_ and *O*_*j*_ was considered. Other atomic pair combinations are not included in the Lennard-Jones interactions within the TIP4*P*/2005 water model. Attractive and repulsive electrostatic interactions are computed using Coulomb’s law. The kinetic energy term, *E*_Kin_^*ij*^, is defined based on the relative motion of the two interacting water molecules. For the kinetic energy of the hydrogen atoms *E*_Kin_^H^, the velocity *v*_H_ used corresponds to the most probable velocity of a particle with the mass of hydrogen at the simulation temperature, as described by the Maxwell–Boltzmann distribution. Additionally, the parameter *E*_Kin_^H^ is weighted by the factor λ, which quantifies the contribution of the kinetic energy of the hydrogen atoms to the total energy summation between molecules *i* and *j*.

In addition, since the calculation of these different energies involves pointwise comparisons between two molecules and the analyzed pair may be influenced by the presence of third molecules, a cylindrical region with a parametrized radius *r*_c_ was considered (The choice of *r*_c_ was tested in the range of 0.20–0.32 nm (around σ_OO_ and the first RDF peak), showing no impact on MAE or RMSE, indicating that the network topology is robust to *r*_c_. We therefore adopt *r*_c_ = σ_OO_ = 0.3159 nm, as it corresponds to a well-established physical length scale in the Lennard-Jones potential and also represents the effective size of the oxygen–oxygen interaction core, ensuring reproducibility in future studies and across different systems). The value of *r*_c_ corresponds to the diameter of the oxygen atom inthe TIP4*P*/2005 water model (0.3159 nm).^14^ The central axis of this cylinder coincideswith the line segment connecting the centers of mass of the two analyzedwater molecules (Figure 1). After these considerations, if any molecule is found within theimaginary cylinder, the interaction between the pair of water moleculeslocated at the cylinder’s ends is disregarded. In other words,only interactions that are free from interference by third moleculesare considered valid and can be analyzed according to the interactioncriterion previously defined in eq 3.

**Figure 1:**
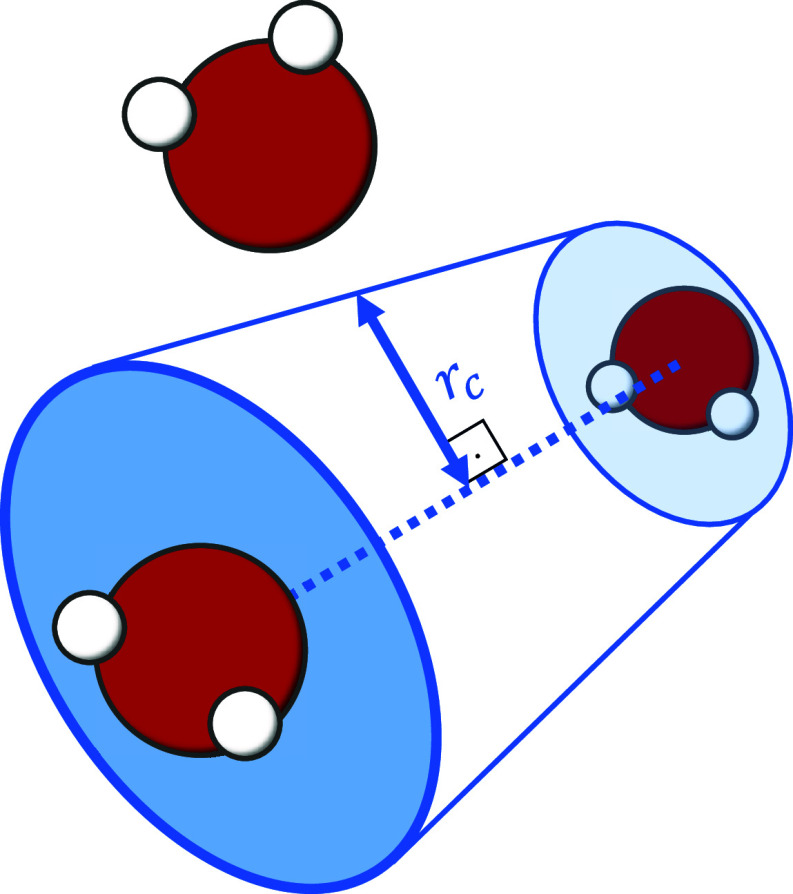
Imaginary interference cylinder.

In order to optimize the algorithms used for calculating the energetic components, particularly with respect to intermolecular distance determination, connections were filtered so that only those involving molecules simultaneously located within a concentric imaginary cube with dimensions equal to 85% of the original cube edge were retained. This filtering aimed to remove interactions that previously occurred due to the periodic boundary conditions but would otherwise be incorrectly treated in the scripts as direct interactions based on the Euclidean distance between the atoms of the considered molecules *i* and *j*.

Furthermore, the construction of networks according to the criterion defined by eq 3, and the imaginary interference cylinder condition was validated by comparing the distribution of the number of connected components, calculated using the NetworkX^32^ library, with the experimental distribution of water cluster sizes under reduced thermodynamic conditions of Tr = 1.04 and Pr = 2.27, as reported by Bernabei et al.^28^ To assess the similarity between both distributions, the MAE and RMSE were used.

### Theoretical Background of Network Topological Properties

If the criterion defined in eq 3 is satisfied, the pair of molecules that interact significantly is recorded in a file containing all the edges that meet the same condition. The resulting list of connections can be interpreted as a graph using the NetworkX^32^ Python library (version 3.5). This library enables efficient and accurate computation of several structural properties of the network, including: (1) network density; (2) average degree; (3) PageRank; and (4) connected components.

The network density ρ of a network quantifies how close the graph is to being completely connected, measuring the ratio between the number of existing edges and the maximum possible number of edges. For an undirected network without loops, it is given by

$$\rho =\frac{2E}{N\left(N-1\right)}$$ 4

where *E* is the number of edges, and *N* is the number of nodes. Network density values close to 1 indicate highly connected networks, in which almost all nodes are connected to each other, while values close to 0 reveal sparse structures.

The degree of a node (*k*_*i*_) corresponds to the number of connections that it establishes with other nodes. The average degree of the network (<*k*>) can be defined in two equivalent ways: by the ratio between twice the total number of edges (*E*) and the total number of nodes (*N*), or by the arithmetic mean of the individual degrees. The formal expression for these calculations is given by

$$\left\langle k\right\rangle =\frac{2E}{N}=\frac{\overset{N}{\underset{i=1}{\sum }}k_{i}}{N}$$ 5

The PageRank metric is an indicator that quantifies the relative importance of each node in a network, derived from an algorithm originally developed to rank web pages and define which ones should appear at the top of search results.^33^ In the context of this study, it is used to identify which nodes exert a greater structural influence on the network formed by interactions between water molecules. PageRank estimates this importance by calculating the probability of a random walker visiting each node, taking into account both the number of connections received and the quality of those connections, which directly reflect the topology of the network. Thus, high PageRank values indicate more central nodes that are more likely to be reached by the walker, while low values suggest lower structural relevance. This metric will be applied in the methodology as a criterion for comparing centrality, the role of nodes within the studied network, and indirectly evaluating the topological similarity between different networks.

The analysis of connected components was performed through the distribution of their sizes, which was fitted to a power function, given by

$$n_{c}\left(s\right)=A\cdot s^{b}$$ 6

From this fitting, the parameters *A* and *b* were obtained, corresponding respectively to the number of isolated water molecules (or nodes) and to the power-law exponent, which describes the decay rate of the frequency of connected components as their size (s) increases.

Based on the different properties of the interaction network, the existence of statistically significant differences among these properties was assessed through *p* value calculations, comparing the distinct thermodynamic conditions previously simulated by molecular dynamics. The *p* values were computed using a Python algorithm with the pandas (version 2.2.2)^34,35^ and SciPy (version 1.16.2)^36^ libraries.

### QTAIM Calculations

For each simulation with a density error below 5.0%, three connected components representative of the types 2:Linear, 3:Linear, and 4:Linear were selected. This selection was performed through principal component analysis (PCA), using as input variables: (1) the sum of the bond lengths within the connected component, (2) the standard deviation of these bond lengths, (3) the mean interaction energy among the molecules within the connected component, and (4) the standard deviation of these interaction energies. Subsequently, the connected components chosen as representative of each type were those located closest to the mean distribution in the sample space defined by the principal components (PCs).

After selecting the representative connected components for each type and thermodynamic condition, these components were characterized through an QTAIM analysis performed using the ORCA (version 5.0.4)^37,38^ and Multiwfn (version 3.7)^39,40^ programs. The wfn files were obtained from DFT calculations carried out at the B3LYP-ZORA level of theory with the ZORA-def2-TZVP basis set and the D4 empirical dispersion correction.

Finally, at the end of the QTAIM calculations, the following topological properties were obtained: (1) total electron density, (2) Laplacian of the electron density, (3) Lagrangian kinetic energy, *G*(*r*), (4) Hamiltonian kinetic energy, *K*(*r*), and (5) potential energy density, *V*(*r*). Subsequently, the existence of statistically significant differences among these topological interaction properties across the different simulations was evaluated through p-value calculations using the pandas (version 2.2.2) and SciPy (version 1.16.2) libraries.

## Results and Discussion

### Validation of Simulation Parameters and Establishment of Criteria for Interaction between Water Molecules

In order to validate the parameters used in the molecular dynamics simulations, the RDFs between the atomic pairs HH, OH, and OO were calculated and compared with experimental data available under the same thermodynamic conditions (Tr = 1.04; Pr = 2.27).^28^ The agreement between the positions and intensities of the main peaks provides a direct measure of the interaction potential’s ability to reproduce the local structure of the supercritical fluid. The comparisons presented in Figure 2 show that the adopted model initially adequately describes the short-range structural correlations under supercritical conditions, thus validating the parameters used in the simulations.

**Figure 2:**
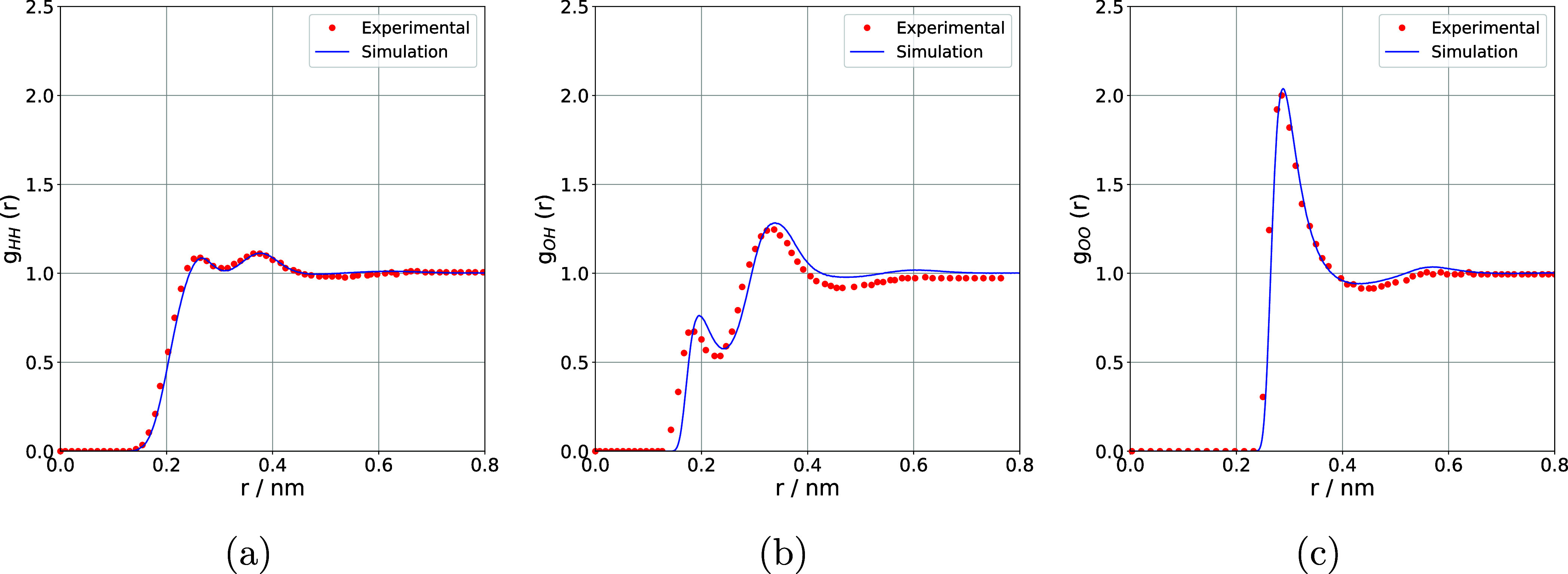
RDFs for atomic pairs (a) HH, (b) OH, and (c) OO of water under supercritical conditions (Tr = 1.04; Pr = 2.27). The simulated curves (blue lines) are compared to experimental data (red dots) obtained under the same thermodynamic conditions.

Furthermore, as shown in Figure 2, the RDFs showed excellent agreement between the simulated results and the experimental data. The correlations were particularly high for the HH (Figure 2a) and OO (Figure 2c) pairs, with *R* ^2^ values of 0.9966 and 0.9854, respectively, demonstrating that the model reproduces the structural characteristics of these interactions with high accuracy. It should be noted that the closer the *R* ^2^ value is to 1, the better the fit between the simulated and experimental data.^41^ Even for the OH pair (Figure 2b), the *R* ^2^ value of 0.9554 indicates a robust linear relationship between the simulated and experimental profiles despite the small horizontal displacement between both RDFs.

Complementarily, the values of *RMSE* and *MAE* reinforce the quality of the fit, whereby the lower the *RMSE* and *MAE* metrics, the greater the accuracy of the simulation in predicting the experimental data.^42^ The RMSEs obtained were low, with 0.0127, 0.0430, and 0.0327 for the HH (Figure 2a), OH (Figure 2b), and OO (Figure 2c) pairs, respectively. In addition, the MAE values were also low, with 0.0074, 0.0331, and 0.0108 for the HH (Figure 2a), OH (Figure 2b), and OO (Figure 2c) pairs, respectively. These results, together with the *R* ^2^ values, indicate that the simulations consistently reproduce the local structures of water under the conditions investigated, confirming the reliability of the parameters adopted in molecular dynamics.

In the same simulation, a representative frame was selected using PCA. Based on the intermolecular interaction criterion defined by eq 3, and considering *E*_Kin_^H^ = 5.60 kJmol^–1^ obtained by the Maxwell–Boltzmann distribution, interaction networks were constructed for different values of λ. The value of λ that minimized the error between the distribution of connected component sizes and the experimental distribution of connected components^28^ was λ = 0.655, yielding *MAE* = 0.004821 and *RMSE* = 0.016121 (Figure 3).

**Figure 3:**
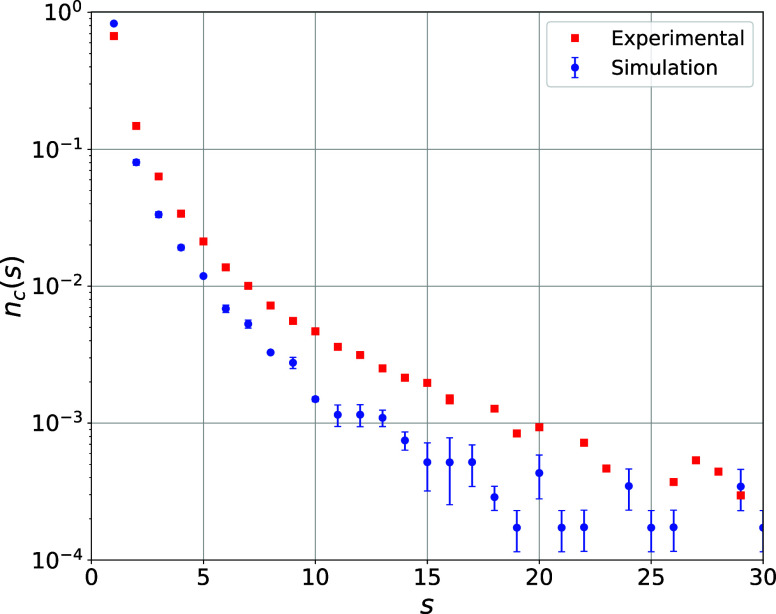
Comparison of connected component size distributions from network model to experimental data of Bernabei et al.^28^ for condition (Tr = 1.04; Pr = 2.27). Reprinted (Adapted or Reprinted in part) with permission from Bernabei, M.; Botti, A.; Bruni, F.; Ricci, M. A.; Sopper, A. K. Percolation and three-dimensional structure of supercritical water. *Phys. Rev. E* **2008**, 78. Copyright 2008 American Physical Society.

In this context, parameter λ acts as an effective scaling factor for the contribution of the kinetic energy of hydrogen atoms to the intermolecular interaction criterion. Rather than indicating a literal reduction in intrinsic kinetic energy, λ should be interpreted as a phenomenological parameter that accounts for constraints and correlations not explicitly included in the paired energy terms, such as the restriction of hydrogen motion due to the covalent bond (O–H) and cooperative effects in hydrogen bond networks.

The optimized value (λ = 0.655) indicates the reduced mobility of hydrogen in systems with hydrogen bonds. A similar behavior was reported by Meier et al., who attributed this effect to intra- and intermolecular interactions, particularly in symmetric hydrogen bonds under high pressure.^43^

Continuing the process of validating the parameters of the molecular dynamics simulations, this stage consisted of performing simulations under 15 different thermodynamic conditions, with the aim of comparing their respective densities with experimental values.^28^ The conditions of simulations were validated by comparing the simulated densities to the experimental values reported in the NIST Webbook, as shown in Table 1. For the continuation of the proposed methodology, the supercritical conditions that presented an error equal to or lower than 5.00% were selected. This set included all simulations with Tr = 1.05 and Tr = 1.10, as well as those with Pr = 3.00, 3.25, and 3.50 at Tr = 1.15, resulting in a total of 13 thermodynamic conditions with errors less than or equal to 5.00%. The validation of thermodynamic conditions was performed exclusively using pure water systems. Given the relatively low ionic concentration and the absence of reliable experimental density data for aqueous chloride solutions under supercritical conditions. Therefore, the validation based on pure water was considered adequate for the continuation of the proposed methodology.^44^

**Table 1:** Conditions of the Simulations

	GROMACS		Density/kg m^–3^		
Condition	Temperature/K	Press/bar	Sim.	Exp.	Error (%)
1	679.451	551.780	584.852	581.043	0.656
2	679.441	606.894	601.227	599.995	0.205
3	679.448	662.010	615.569	616.043	0.077
4	679.454	717.096	628.451	630.040	0.252
5	679.449	772.485	640.036	642.582	0.396
6	711.805	551.690	505.245	485.125	4.147
7	711.814	606.844	529.353	516.209	2.546
8	711.815	661.918	549.482	540.903	1.586
9	711.809	716.868	566.706	561.344	0.955
10	711.809	772.345	581.943	578.963	0.515
11	744.167	551.574	419.497	377.487	11.129
12	744.171	606.735	450.922	421.276	7.037
13	744.159	661.966	478.421	456.273	4.854
14	744.162	717.228	501.073	484.784	3.360
15	744.152	772.460	520.543	508.587	2.351

The largest error observed in simulations 11 and 12 is directly related to the higher compressibility (eq 7) of the system under these thermodynamic conditions. At high temperatures and lower pressures, the fluid has a higher β_T_ value, given by,

$$\beta_{T}=-\frac{1}{V}{\left(\frac{\partial V}{\partial P}\right)}_{T}$$ 7

indicating that small pressure variations result in relatively large volume variations. Since density is inversely proportional to volume, small inaccuracies in the effective pressure of the ensemble or in the description of intermolecular interactions lead to significant variations in the simulated density. Thus, the higher compressibility amplifies model errors and statistical fluctuations, resulting in higher deviations from the experimental values.

### Energetic Characterization of Molecular Dynamics Simulations

The evaluation of molecular dynamics simulations in this subsection is mainly done in terms of kinetic and potential energies, a choice aligned with the central objective of this work: to construct and analyze networks of intermolecular interactions defined by an energetic criterion. These quantities are therefore the most relevant descriptors, as they underpin intermolecular interactions and network topology, as established in this work, based on eq 3. This approach follows the literature that treats water and supercritical fluids as complex systems described by interaction networks, in which molecular dynamics serves mainly to generate representative configurations without extensive detailing of temporal dynamics, as demonstrated in previous studies.^6,45^ Thus, the energy-based discussion provides a more direct description that is consistent with the scope of this study, consistently integrating molecular dynamics simulations with the topological analysis of intermolecular interactions.

At the end of the simulations, the kinetic and potential energies of each system were calculated and are summarized in Table S1 (From now on, figures and tables in the Supporting Information (SM) will be labeled as Figure S*X* and Table S*X*, respectively, where *X* denotes the item number). For both systems studied, pure water and water with Cl^–^, an increase in temperature led to higher kinetic energy and less negative potential energy values. Likewise, increasing pressure affected both systems in a similar manner, mainly increasing the magnitude of the potential energy, while having no significant effect on the kinetic energy. The introduction of Cl^–^ ions into the aqueous systems had a practically irrelevant effect on kinetic energy but caused a substantial increase in potential energy. This increase reflects the formation of new intermolecular interactions, both between water molecules and between water molecules and chloride ions, regardless of the temperature and pressure conditions adopted.

Table 2 summarizes the relative variations in kinetic and potential energies for both systems. Relative standard deviations across different temperatures and pressures were computed to quantify the energetic sensitivity of each system to these thermodynamic parameters. These results provide insights into the influence of chloride ions on the energetic fluctuations of the aqueous system.

**Table 2:** Relative Standard Deviations of Kinetic and Potential Energies for Pure Water and an Aqueous Solution Containing Cl^–^ Ions

System	Energy	Standard deviation across temperature (%)	Standard deviation across pressure (%)
Water	Kinetic	4.546	1.256
	Potential	8.697	1.362
Water + Cl^–^	Kinetic	4.586	1.254
	Potential	5.710	0.874

From Table 2, it is possible to extract some relevant insights into how the thermodynamic conditions adopted in this work influence the energies of the system. It can be observed that among the intensive variables analyzed, temperature plays a more significant role in differentiating between the simulated conditions than pressure, promoting greater variations in both kinetic energy and potential energy, both in the neutral pure water system and in the system containing Cl^–^ ions.

The kinetic energy exhibits very similar standard deviations for both pure water and the aqueous solution containing Cl^–^ ions (≈4.56% with respect to temperature and ≈1.25% with respect to pressure). These results indicate that the presence of ions has a negligible influence on the average thermal behavior of water molecules, since kinetic energy is directly related to the system temperature.^46^ In contrast, the effect of Cl^–^ ions is much more pronounced on the potential energy, suggesting that their presence stabilizes intermolecular interactions and reduces the sensitivity of this quantity to variations under the thermodynamic conditions. The addition of Cl^–^ ions can modify the microscopic structure of water by altering intermolecular distances and the balance between attractive and repulsive interactions.^47^ This behavior arises primarily from ion–dipole interactions, which are less sensitive to small pressure variations than the intermolecular interactions between water molecules. As a result, pressure becomes a less effective factor in modifying the local structure of the system.

### Network Topological Properties

Although classical topological descriptors commonly employed in static complex networks, such as the clustering coefficient or shortest path length, are widely used to characterize equilibrium or time-averaged structures, they are not fully suited to describe systems dominated by highly transient interactions, such as supercritical water. In this regime, intermolecular interactions are continuously formed and disrupted due to intense thermal motion, leading to networks that evolve rapidly over time and exhibit strong spatial and temporal heterogeneity. Therefore, in the present work we adopt a set of topological parameters typically associated with dynamic complex networks, which are particularly sensitive to changes in connectivity, interaction persistence, and node relevance. This alternative approach provides a more appropriate framework for linking thermodynamic conditions to the global organization of intermolecular interactions while remaining consistent with the inherently dynamic nature of the system under investigation.

The topological metrics of an interaction network play an essential and novel role in characterizing its structural and dynamic properties, allowing for a quantitative understanding of how its elements are organized and interact.^48^ In this perspective, the density of a network represents the ratio between the number of connections that actually exist and the maximum number of possible connections in a complete graph with the same number of nodes. From our results, we observed a slight decrease in connection network density with increasing temperature (Figure 4). This behavior can be understood by considering positive energy terms, such as *E*_Kin_^*ij*^ and *E*_Kin_^H^, which are directly proportional to the system temperature. As thermal energy increases, these terms grow, making it more difficult for the intermolecular interaction criterion defined by eq 3 to be satisfied. Consequently, the number of effective collisions and attractive interactions between particles decreases, which explains the reduction in the connection network density.

**Figure 4:**
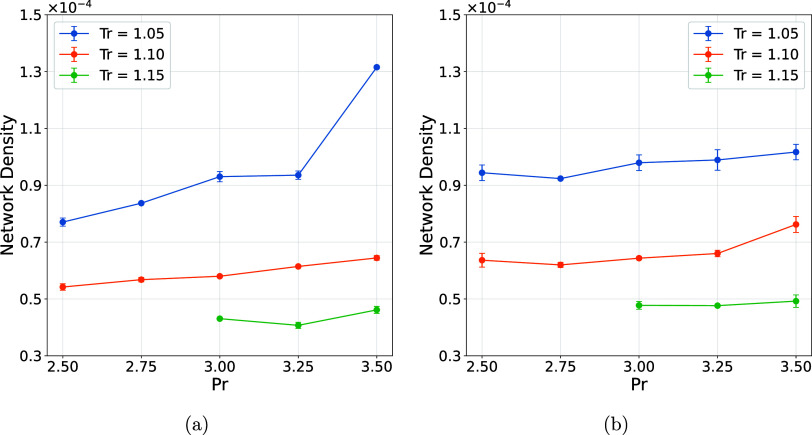
Intermolecular network density considering all water molecules. (a) Water and (b) water + Cl^–^.

In contrast, Figure S1 shows the network density calculated only for molecules that actually participate in interactions (excluding those with zero connection degree), revealing an increase in network density with rising temperature. This reversal of trend results from the progressive exclusion of isolated molecules, which reduces the number of nodes considered and results in a network composed of a relatively more connected subset; thus, even if the absolute number of interactions *E* remains constant, the term *N*(*N* – 1) in the denominator decreases, causing the network density to increase, as evidenced by eq 4.

Since the network density metric does not distinguish the intensity or duration of interactions, considering only their count within a single frame, it is not suitable for assessing the transient nature of intermolecular interactions. Therefore, although the network density decreases with temperature, this effect primarily reflects the reduction of temporary contacts rather than changes in the strength, structural organization, or qualitative character of the effective interactions. Additional insight into this behavior can be obtained from the mean PageRank metric (Figure 5), which captures the relative importance of each node based on its connectivity and the structure of its neighboring links. Disregarding monomeric molecules for network assembly, at lower temperatures, the interaction network is more homogeneous and static, leading to a relatively uniform distribution of PageRank values among molecules. As the temperature increases, however, the enhanced molecular mobility and rapid formation and disruption of interactions create a more heterogeneous and dynamic network.

**Figure 5:**
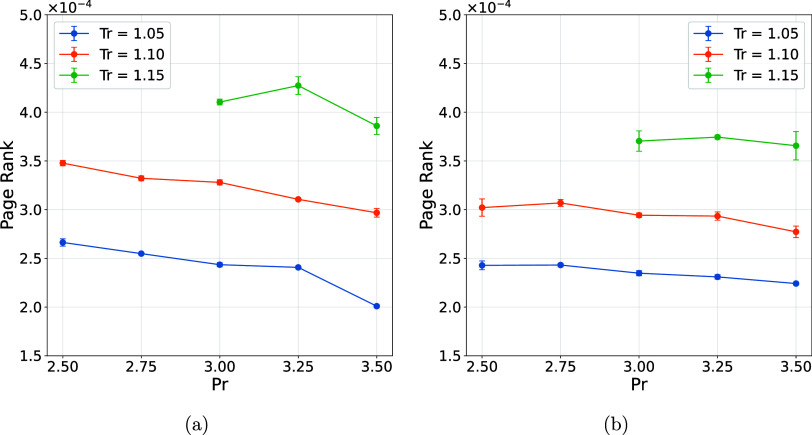
PageRank mean considering only interacting water molecules. (a) Water and (b) water + Cl^–^.

Therefore, the decrease in network density occurring simultaneously with the increase in the average PageRank indicates that these interactions are not uniformly distributed in time or space but instead tend to concentrate around transient connectivity centers. This behavior reinforces the interpretation that, at higher temperatures, the intermolecular network is characterized by a larger number of fleeting and spatially heterogeneous interactions, reflecting the ephemeral and fluctuating nature of the liquid structure.^49,50^

In contrast to the temperature, which strongly disrupts intermolecular connectivity by increasing kinetic energy and promoting the rupture of transient interactions, pressure acts primarily by modulating molecular proximity. An increase in pressure reduces intermolecular distances, enhancing the probability of interaction according to the energetic criterion defined in eq 3. This effect is reflected in a slight increase in the number of interactions (Figure 4) when compared at a constant temperature. In this sense, temperature governs the disruption and renewal dynamics of the network, while pressure mainly tunes the spatial constraints under which these interactions occur. The interplay between these variables highlights that, although both thermodynamic parameters influence network topology, temperature is the dominant factor driving topological fragmentation, whereas pressure plays a secondary but nonnegligible role in promoting connectivity.

The average degree metric was also computed (Figure S2), revealing that the mean degree of the water–water interaction network decreases with increasing temperature but slightly increases after the addition of chloride ions. The decrease with temperature indicates that, despite the greater number of transient interactions reflected in the network density, each water molecule tends to engage in fewer simultaneous water–water connections on average. This reflects the fragmentation and dynamism of the interaction network at higher temperatures (see Figures S3–S15 in the Supporting Information).

Conversely, the slight increase in the average degree observed after the addition of chloride ions can be attributed to the formation of water–ion interactions, which draw water molecules closer together in the first solvation shell around the ions. This spatial proximity facilitates secondary water–water interactions among the solvating molecules, thereby increasing the mean number of water–water connections present in the interaction network.

This effect becomes even more evident when considering the reduction in the number of monomeric water molecules following chloride ion addition, a trend reflected by parameter A in eq 6 and shown in Figure 6. As the number of monomeric molecules—i.e., those with zero connectivity degree—decreases, the effective number of nodes in the topological network consequently increases. This expansion reduces the relative relevance that certain molecules exhibited prior to the introduction of chloride ions, as evidenced by the lower PageRank values when comparing the water and water + Cl^–^ systems. In other words, the presence of the chloride ion redistributes the network connectivity, diluting the centrality that was previously concentrated in specific molecules. Moreover, the increase in the power-law exponent b associated with the distribution of connected component sizes, as shown in Figure 7, further supports this interpretation, indicating that the presence of chloride ions leads to more frequent formation of larger connected components compared with the pure water system.

**Figure 6:**
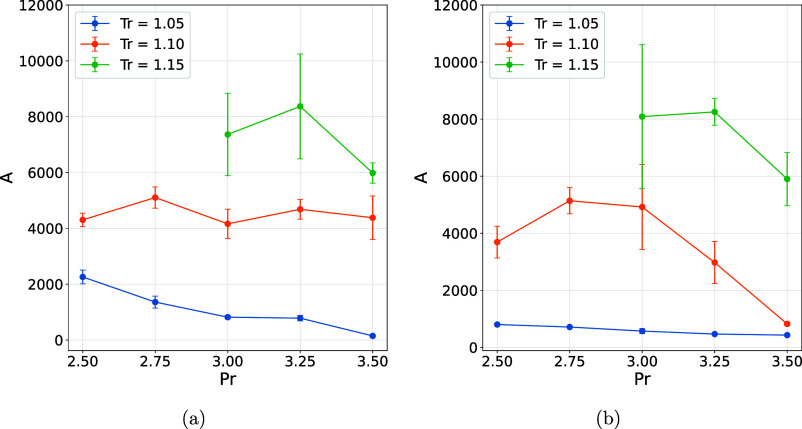
Parameter A. (a) Water and (b) water + Cl^–^.

**Figure 7:**
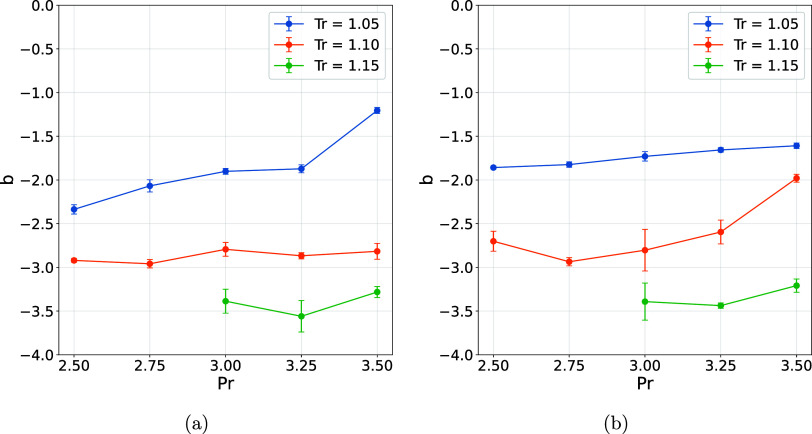
Parameter B. (a) Water and (b) water + Cl^–^.

Analysis of the distribution of connected component sizes revealed a systematic dependence of the fitting parameters represented by eq 6 on the temperature. It was observed that as the temperature increases, parameter A grows, indicating a greater predominance of monomeric components and therefore more pronounced fragmentation of the network (Figure 6). Simultaneously, exponent b becomes more negative, reflecting a faster decay in the frequency of components as size increases, which highlights the reduction in the formation of larger aggregates under high thermal conditions (Figure 7). Collectively, these findings indicate that elevating the temperature diminishes the connectivity of intermolecular interactions, thereby promoting the dissociation of connected components and substantially modifying the global structural organization of the system (see Figures S16–S28).

The topological properties of the network of intermolecular interactions directly reflect the thermodynamic state of the system, acting as a collective descriptor of its structural organization. Increasing the temperature by intensifying molecular kinetic energy increases the topological fragmentation of intermolecular interactions, leading to more fragmented networks, with a lower average degree, lower overall network density, and distributions of connected components dominated by smaller aggregates. In contrast, pressure variations tend to act more subtly, favoring molecular proximity and the persistence of connections without significantly altering the local nature of interactions. Thus, while thermodynamic properties control the balance between thermal energy and attractive interactions, topological metrics translate this balance into global connectivity patterns that are sensitive to thermal fluctuations and capable of capturing collective structural changes.

### Intermolecular Interactions Properties

Although network topological descriptors capture how intermolecular interactions are globally organized and respond to thermodynamic variations, a detailed analysis of intermolecular interaction properties is essential to understand how these collective patterns are sustained at the local electronic level. To define a statistically representative set of molecular motifs for the calculation of electronic properties, we first analyzed the distribution of connected component sizes obtained from the interaction networks. On average, connected components of size 2 correspond to 52.11% of the total, with a standard deviation of 5.77% between the different simulations. Size 3 components represent, on average, 20.77%, with a standard deviation of 1.44%, while size 4 components correspond to 10.28%, with a standard deviation of 1.25%. These results show that connected components of sizes 2, 3, and 4 constitute most of the observed structure and show moderate variations between the simulations. Thus, in the QTAIM calculation, the analysis is primarily directed at these components, as they are statistically more representative and therefore more suitable for the topological characterization of the system.

To understand the electronic features underlying intermolecular interactions in water and in aqueous ionic environments, QTAIM has been performed on snapshots from molecular dynamics simulations. This analysis provides insights into the distribution of electronic density and energy contributions at bond critical points, allowing the characterization of intermolecular interactions.

The total electron density ρ­(**r**) is obtained by summing the contributions of all natural orbitals, each weighted by its corresponding occupation number, thus describing how electrons are distributed at the critical point under analysis. From this density, different energetic quantities can be defined. The Lagrangian kinetic energy *G*(**r**) corresponds to the always positive form of the kinetic energy density, derived from the gradient of the orbitals. In contrast, the Hamiltonian kinetic energy *K*(**r**), based on the Laplacian operator, may assume negative values, depending on the local behavior of the electron density. Using these two components, one determines the potential energy density *V* (**r**), given by *V* (**r**) = – *G*(**r**) – *K*(**r**), which reflects the local potential at the critical point and plays an important role in analyses within the QTAIM framework, such as in the characterization of hydrogen bonds. Additionally, the Laplacian of electron density ∇^2^ρ­(**r**) reveals regions of electron density concentration (negative values) or depletion (positive values), providing essential information about the nature of chemical interactions in the system.

Following the criteria described by Kumar et al. for classifying interactions according to QTAIM, which use electronic density ranges ρ­(**r**), sign of ∇^2^ρ­(**r**), local total energy *H*(**r**), ratio [−*G*(**r**)/*V*(**r**)], and relative contribution of *G*(**r**) to distinguish noncovalent, partially covalent, and covalent interactions,^51^ we evaluated all BCPs present in the two analyzed systems, with 238 BCPs for the pure water system and 237 for the water + Cl^–^ system. Most of the BCPs identified in our study fell into the category of noncovalent interactions, both in the pure water system and in the water + Cl^–^ system. However, in both systems, approximately 10.50% of the BCPs were classified as partially covalent interactions, occurring exclusively in the connected components 3:Linear and 4:Linear. In the pure water system, these components presented 11.69 and 13.11% of partially covalent interactions, respectively, while in the water + Cl^–^ system, the corresponding values were 10.26 and 14.17%. No covalent interactions were identified in either system.

Furthermore, as the number of water molecules in the clusters increases, both the electron density ρ­(**r**) and the potential energy density *V*(**r**) at the bond critical points associated with intermolecular interaction also rise, with the *V*(**r**) becoming dominant in larger clusters. This behavior reflects the strengthening of the intermolecular interactions, classified by Kumar et al. as partially covalent, evidenced by the higher electron density and the more negative potential energy values at the BCPs.^51^ These trends are fully consistent with the analysis of Guevara-Vela et al., who demonstrated that the electron density at the bond critical point of the hydrogen bond increases monotonically with cluster size and that the exchange energy and delocalization indices also grow,^52^ indicating an increase in the covalent nature of the interaction between oxygen and hydrogen atoms. Their results further show a systematic strengthening of the intermolecular interaction energies as additional water molecules are incorporated.

The absence of statistically significant differences in all electronic properties analyzed (*p* value ≥ 5.00 × 10^–2^) (Table 3) indicates that the temperature and pressure variations considered do not promote detectable changes in the overall electronic description of intermolecular interactions. In particular, fundamental quantities such as total electron density, its Laplacian, and the kinetic and potential components of energy exhibit remarkable statistical robustness. This behavior suggests that the average pattern of electronic redistribution associated with intermolecular interactions remains essentially unchanged throughout the thermodynamic space investigated.

**Table 3:** Values of *p* Resulting from a Statistical Comparison of the Structural and Electronic Properties of the Water and Water + Cl^–^ Systems Evaluated under Different Temperature and Pressure Conditions

	*p* value	
properties	Water	Water + Cl^–^
Network density	6.91 × 10^–20^	9.75 × 10^–15^
Average degree	5.21 × 10^–29^	2.59 × 10^–18^
PageRank mean	7.99 × 10^–24^	3.26 × 10^–16^
A	7.96 × 10^–8^	4.28 × 10^–7^
b	8.08 × 10^–17^	8.21 × 10^–14^
Total electron density	6.75 × 10^–1^	5.64 × 10^–1^
Laplacian of electron density	8.43 × 10^–1^	5.79 × 10^–1^
Lagrangian kinetic energy *G*(**r**)	7.35 × 10^–1^	5.42 × 10^–1^
Hamiltonian kinetic energy *K*(**r**)	4.17 × 10^–1^	5.34 × 10^–1^
Potential energy density *V*(**r**)	6.55 × 10^–1^	5.27 × 10^–1^

### From Local Interactions to Global Structure: A Multiscale Analysis

To evaluate the impact of the chloride ion on the electronic structure of water, as well as the influence of different thermodynamic conditions on the topological properties, we calculated the *p* values for a set of QTAIM-derived topological descriptors for both pure water and water + Cl^–^ systems (a *p* value <5.00 × 10^–2^ denotes a statistically significant difference between the systems, while a *p* value ≥ 5.00 × 10^–2^ suggests that any observed difference may be attributed to chance and is therefore not statistically significant). These values were compared across multiple simulations performed under distinct temperature and pressure conditions to assess whether thermodynamic variations induce systematic changes in the properties analyzed. Table 3 summarizes the results, allowing direct observation of how the introduction of Cl^–^ and the modification of the thermodynamic state affect parameters associated with the electron density distribution and the topological connectivity of the hydrogen bond network. Taken together, these data provide quantitative insight into how both the ion and the surrounding thermodynamic environment modulate the local organization of water.

It is observed that all network topological properties investigated in this study, namely, network density, Average degree, PageRank mean, and the parameters A and b, exhibit extremely low *p* values (*p* value < 5.00 × 10^–2^) in both systems. These results indicate statistically significant differences in the structural characteristics of the intermolecular interaction network when thermodynamic conditions are varied, highlighting the high sensitivity of network metrics to changes in thermodynamic parameters in both systems.

In contrast, the local electronic properties obtained from QTAIM analysis, including total electron density, the Laplacian of the electron density, Lagrangian kinetic energy *G*(**r**), Hamiltonian kinetic energy *K*(**r**), and potential energy density *V*(**r**), display high *p* values (*p* value ≥ 5.00 × 10^–2^) for both systems. This indicates that within the level of theory employed, there are no statistically significant differences in these local electronic properties as a function of the thermodynamic conditions considered in the present work.

This behavior points to the robustness of intermolecular interactions in the face of the thermal and compressive fluctuations considered, even in the presence of the Cl^–^ anion. Thus, the results suggest that the electronic mechanisms underlying the water–water interaction are governed mainly by local effects and are not sensitive, on average, to the thermodynamic conditions explored. This finding reinforces the idea that the average electronic description of these interactions is intrinsically stable, supporting the transferability of the electronic models used within the range of conditions investigated.

This direct comparison reveals a clear contrast between the topological and local electronic responses of the system: while the global organization of the water–water intermolecular interaction network is strongly affected by thermodynamic conditions and by the presence of the Cl^–^ ion, the electronic properties at the bond critical points remain essentially invariant. These findings suggest that externally induced changes manifest predominantly at a collective and topological level without significantly altering the local electronic nature of the intermolecular interactions. This reinforces the value of complex network approaches as an emerging methodology for detecting collective structural responses that cannot be accessed through local electronic analysis.

## Conclusions

This study systematically investigated the relationship between thermodynamic conditions and the microstructural organization of water in a supercritical regime. To this end, we used a novel approach, employing complex network tools to describe water as an interconnected system of intermolecular interactions. Integrating this strategy with the fundamentals of classical physics and QTAIM, we characterized the topology and dynamics of molecular clusters under different thermodynamic conditions and also evaluated the impact of ion addition on the topological organization and reorganization processes of these networks. Our work thus proposes a new conceptual and methodological framework for analyzing water under supercritical conditions, surpassing traditional descriptions based solely on RDFs or local statistical analyses.

Our results consistently show that thermodynamic conditions determine the overall organization of the network of intermolecular interactions of water in a supercritical regime, while local electronic properties remain essentially unchanged. Molecular dynamics simulations, validated by excellent agreement with RDFs and experimental densities as well as by the size distribution of connected components, allowed us to construct intermolecular networks that quantitatively reproduce the observed structural distribution. Topological analysis indicated that increasing temperature progressively fragments the network, reducing connection network density and average degree and systematically altering the distribution of connected components, reflected in parameters A and b of eq 6. The addition of Cl^–^ ions promotes a distinct reorganization, favoring larger aggregates, reducing the number of isolated molecules and generating slightly more connected networks. Despite these collective changes, QTAIM analysis showed that electronic properties at critical bonding points (electronic density, Laplacian, and energy components) do not vary statistically significantly with temperature, pressure, or the presence of ions. This contrast indicates that the system’s response to thermodynamic perturbations is predominantly collective and topological, rather than local electronic, highlighting the high sensitivity of network metrics as descriptors of the structural organization of water under supercritical conditions.

Although only a single ion concentration was considered in this study, it is expected that different concentrations could influence the observed properties of the network. In particular, higher ion concentrations would increase the ion–ion interactions and collective electrostatic effects, potentially leading to more pronounced changes in the network topology. On the other hand, at lower concentrations, the perturbation introduced by the ions would be weaker, approaching the topology observed in pure water. Future studies could systematically investigate the effect of the ion concentration on these network properties.

The invariance of local electronic properties, given the high sensitivity of topological metrics to thermodynamic conditions, shows that network descriptors are particularly effective tools for integrating microscopic and macroscopic levels of description. The proposed methodology, based on interference-free energy criteria, complex network concepts, and QTAIM analysis, can be directly extended to other supercritical systems, different ionic species, and broader ranges of thermodynamic conditions. The results indicate that predictive models can be improved by prioritizing collective structural parameters, reducing the dependence on high-resolution electronic descriptions. From an applied perspective, the study supports the improvement of understanding and control of processes that employ supercritical water in which the transient organization of the interaction network is decisive.

## Supplementary Material



## Notes

Figures and tables that complement the results discussed in the main text, including kinetic and potential energy values obtained from the simulations; as well as topological properties of the analyzed networks, including the density considering only interacting molecules, average degree considering all molecules, degree distribution of the system nodes, and the distribution of the number of connected components throughout the simulations (PDF)
