# Supplementary material for: Complex Network and QTAIM Analyses Reveal the Dominance of Structural over Electronic Modulation in Supercritical Water under Ion-Free and Ionic Conditions
Source: ACS Omega. 2026 Apr 29;11(18):27513–24. doi: 10.1021/acsomega.6c02186 (PMC13177021; doi:10.1021/acsomega.6c02186)
Supplement: Supplementary file 1 [file ao6c02186_si_001.pdf]

Supplementary Material: Complex Network and QTAIM Analyses Reveal the Dominance of Structural over Electronic Modulation in Supercritical Water under Ion-Free and Ionic Conditions

Artur G. Nogueira,[†] Angélica S. da Mata,[‡] and Teodorico C. Ramalho[*,†,¶]

†*Department of Chemistry, Federal University of Lavras, Brazil*

‡*Department of Physics, Federal University of Lavras, Brazil*

¶*Centre for Basic and Applied Research, Faculty of Informatics and Management, University of Hradec Králové, Czech Republic*

E-mail: teo@ufla.br

Phone: +55 (35) 3829-1522. Fax: +55 (35) 3829-1271

Table S1: Kinetic and potential energies obtained in simulations under different thermodynamic conditions.

Cond.	Tr	Pr	Kinetic Energy (kJ/mol) Water	Kinetic Energy (kJ/mol) Water + Cl^-	Potential Energy (kJ/mol) Water	Potential Energy (kJ/mol) Water + Cl^-
1	1.05	2.50	144794	145243	-218687	-246149
2	1.05	2.75	144791	145225	-221832	-247648
3	1.05	3.00	144793	145217	-224581	-249419
4	1.05	3.25	144794	145209	-227043	-250985
5	1.05	3.50	144793	145200	-229251	-252388
6	1.10	2.50	151688	152249	-196268	-231174
7	1.10	2.75	151690	152222	-200996	-233112
8	1.10	3.00	151691	152196	-204996	-234684
9	1.10	3.25	151689	152179	-208414	-236424
10	1.10	3.50	151689	152160	-211420	-237906
13	1.15	3.00	158583	159209	-184103	-220586
14	1.15	3.25	158584	159176	-188797	-222558
15	1.15	3.50	158582	159151	-192797	-224241

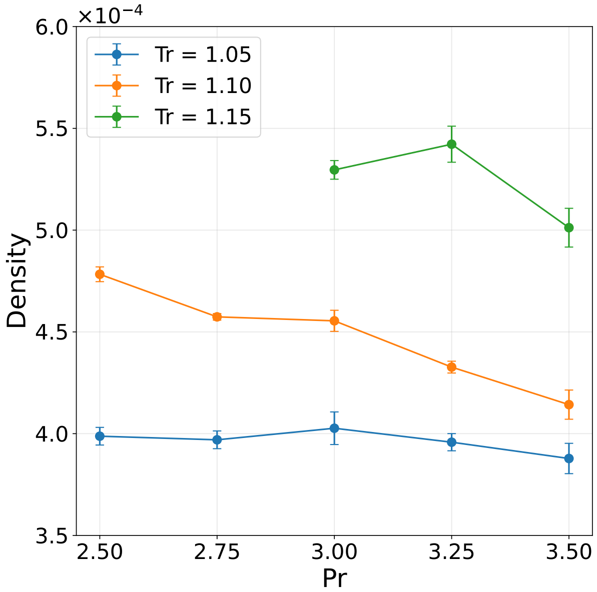

(a)

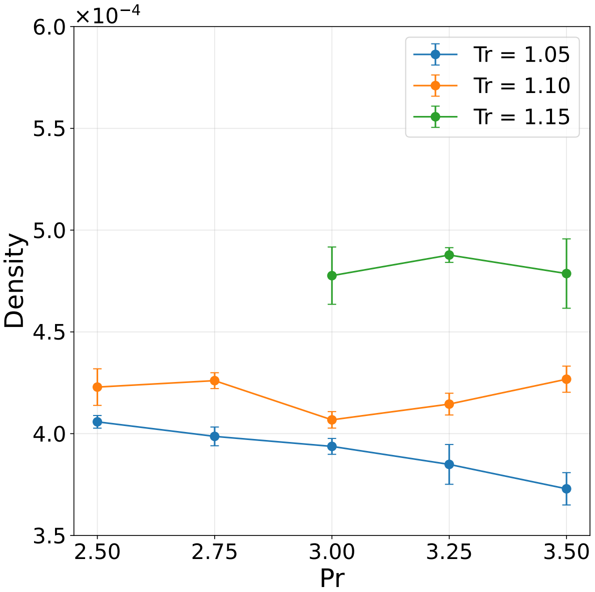

(b)

Figure S1: Intermolecular network density considering only interacting water molecules. (a) Water and (b) Water + Cl^-.

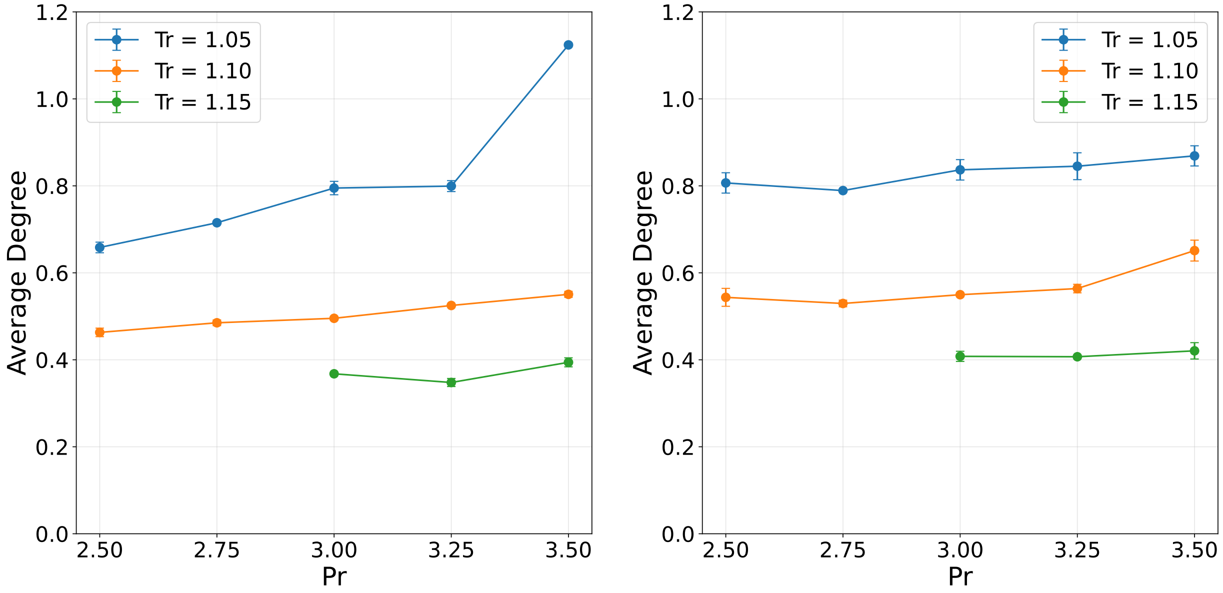

(a) (b)

Figure S2: Average degree of network considering all water molecules. (a) Water and (b) Water + Cl^{-}.

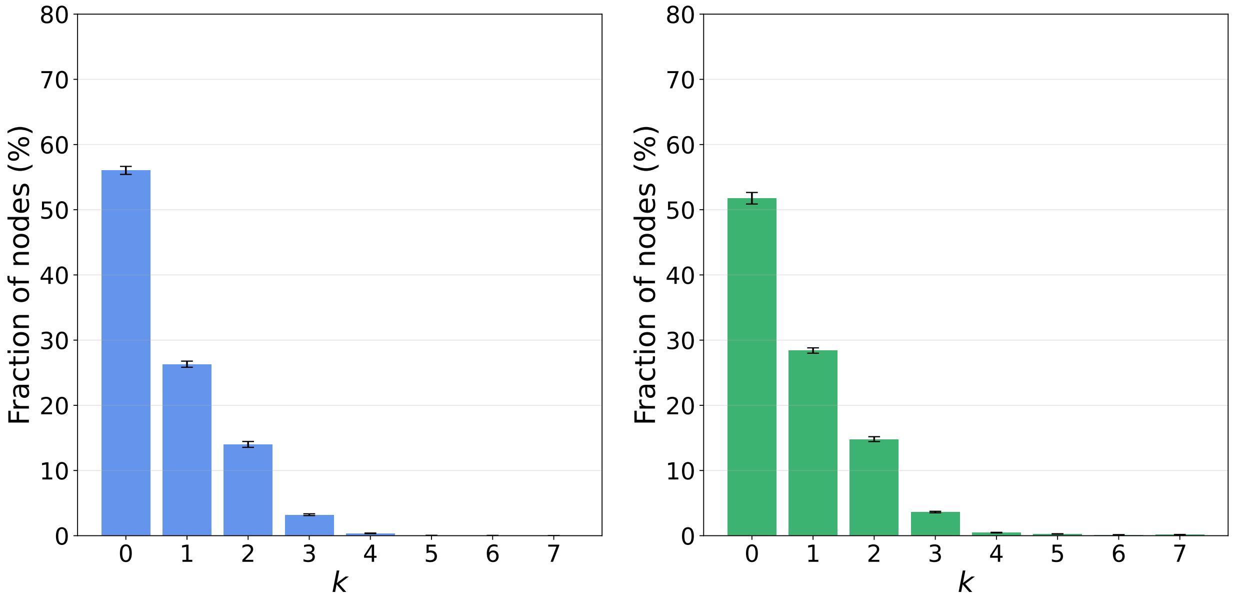

(a) (b)

Figure S3: Intermolecular connectivity distribution under simulated conditions ($Tr = 1.05$; $Pr = 2.50$). (a) Water and (b) Water + Cl^{-}.

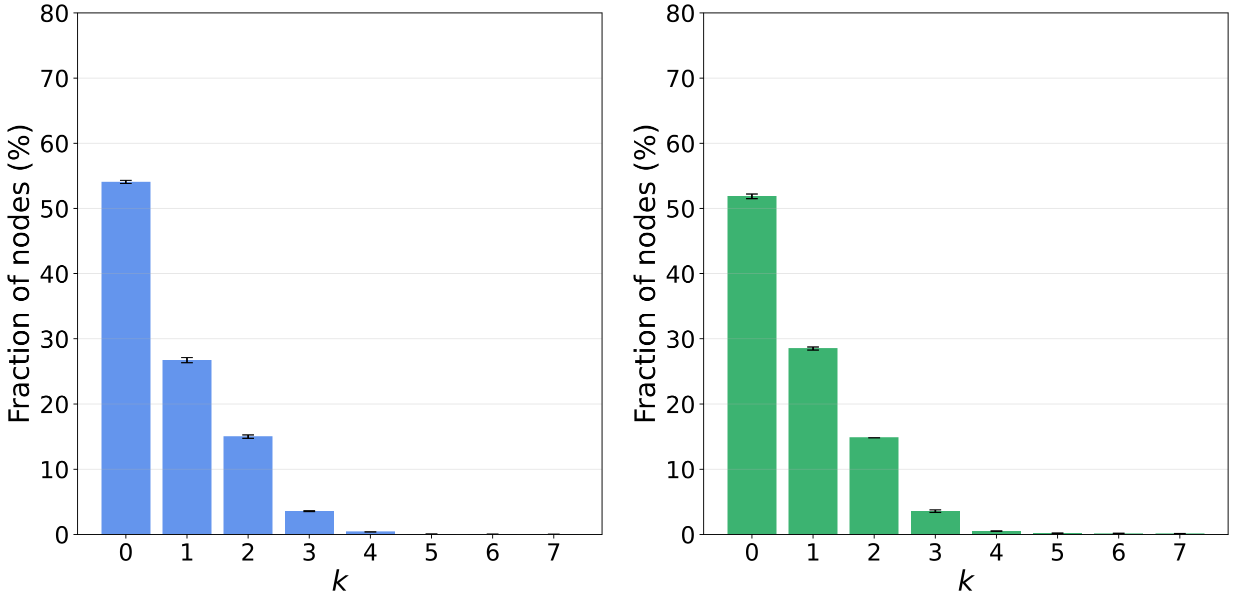

(a) (b)

Figure S4: Intermolecular connectivity distribution under simulated conditions ($Tr = 1.05$; $Pr = 2.75$). (a) Water and (b) Water + Cl^-.

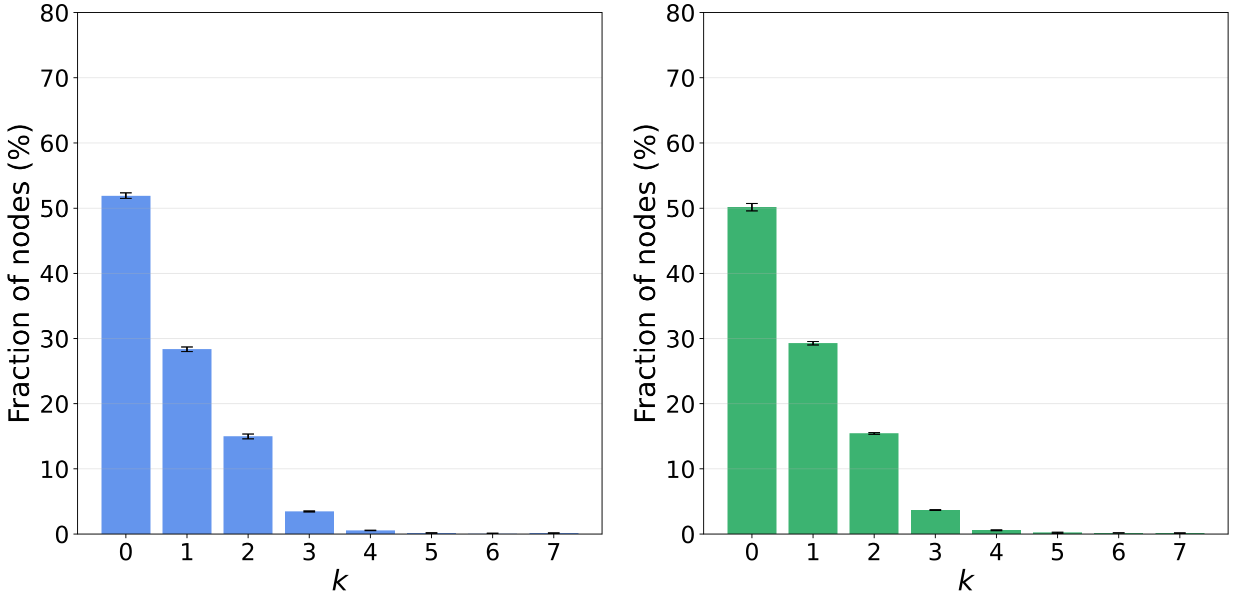

(a) (b)

Figure S5: Intermolecular connectivity distribution under simulated conditions ($Tr = 1.05$; $Pr = 3.00$). (a) Water and (b) Water + Cl^-.

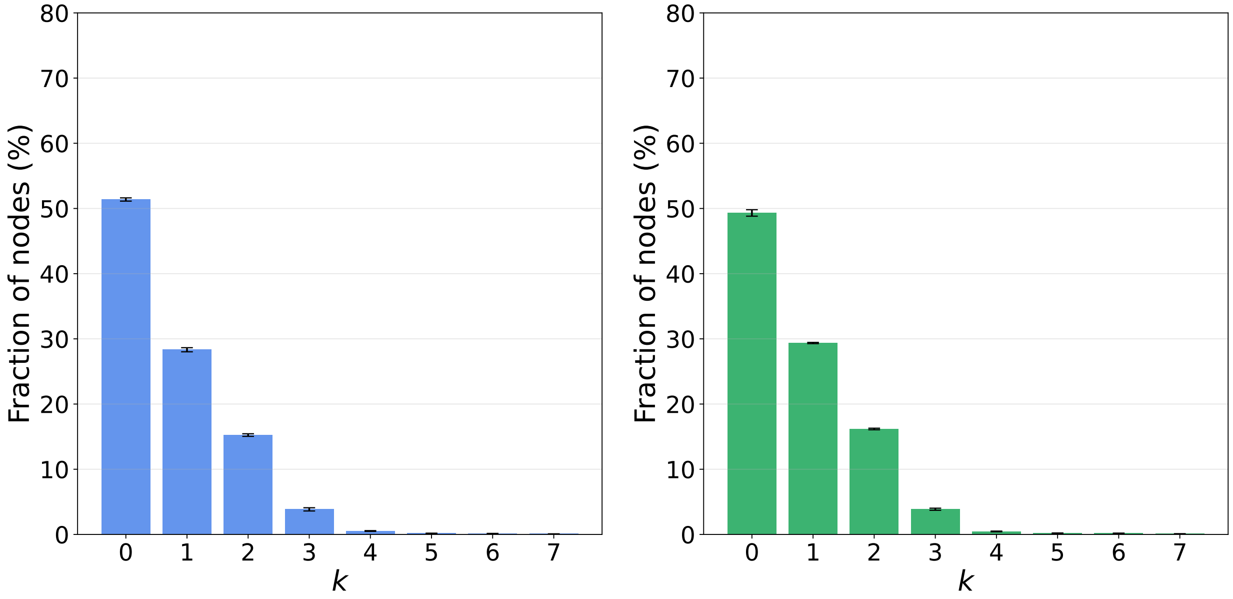

(a) (b)

Figure S6: Intermolecular connectivity distribution under simulated conditions ($Tr = 1.05$; $Pr = 3.25$). (a) Water and (b) Water + Cl^{-}.

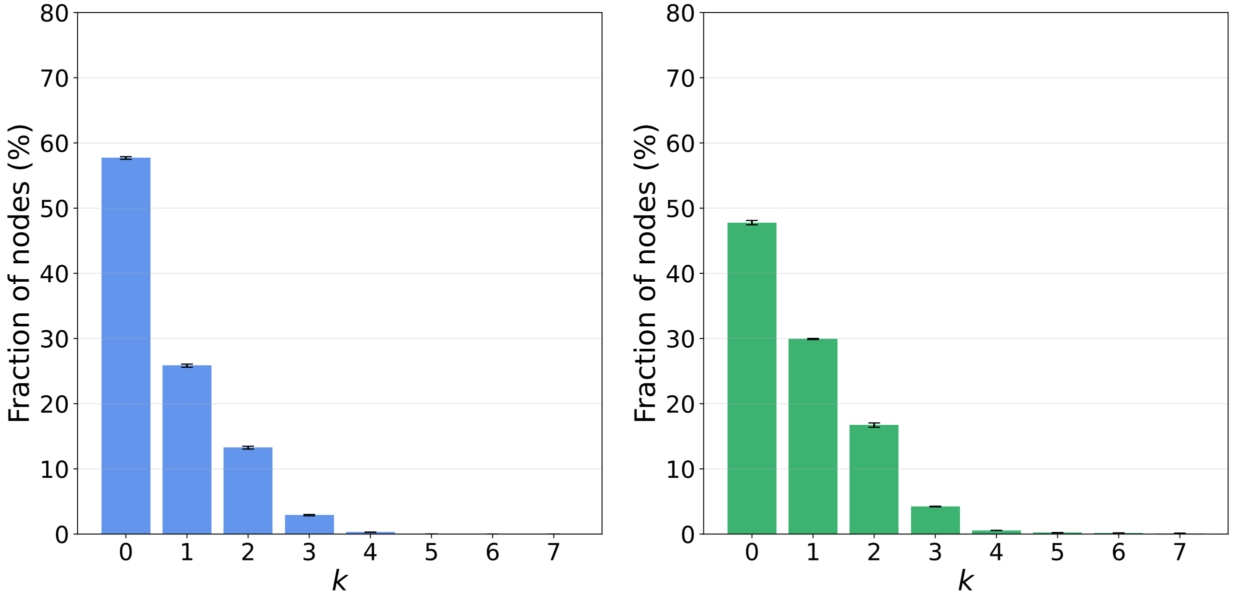

(a) (b)

Figure S7: Intermolecular connectivity distribution under simulated conditions ($Tr = 1.05$; $Pr = 3.50$). (a) Water and (b) Water + Cl^{-}.

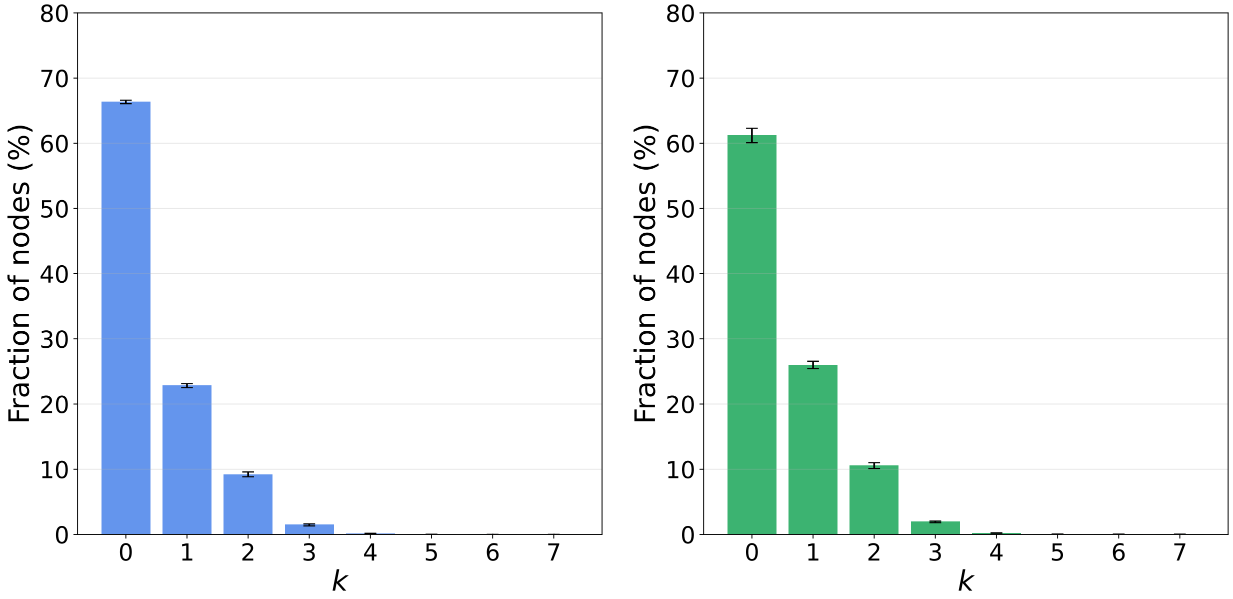

(a) (b)

Figure S8: Intermolecular connectivity distribution under simulated conditions ($Tr = 1.10$; $Pr = 2.50$). (a) Water and (b) Water + Cl^-.

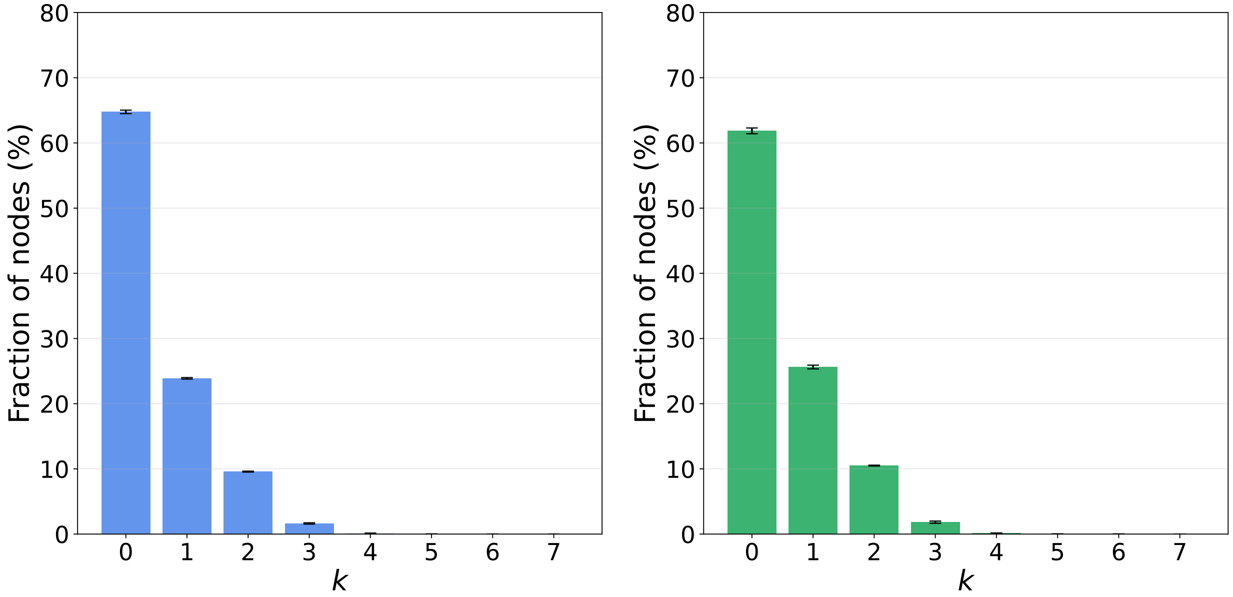

(a) (b)

Figure S9: Intermolecular connectivity distribution under simulated conditions ($Tr = 1.10$; $Pr = 2.75$). (a) Water and (b) Water + Cl^-.

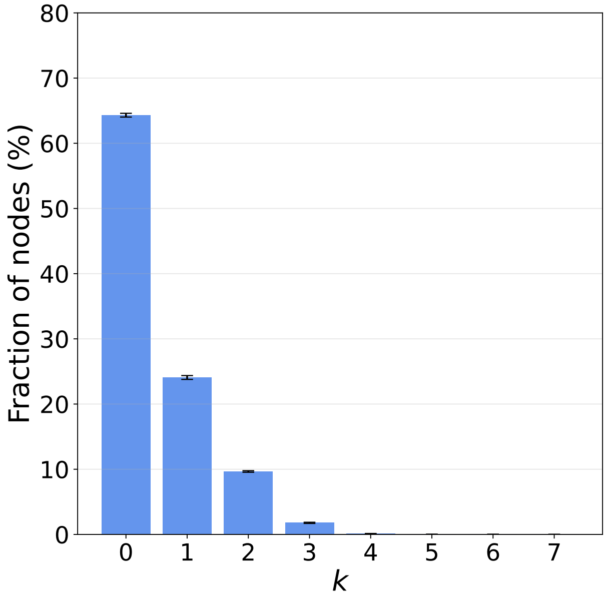

(a)

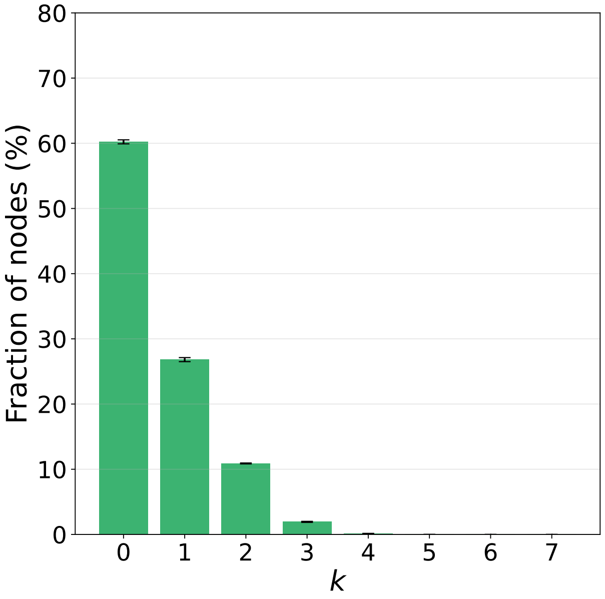

(b)

Figure S10: Intermolecular connectivity distribution under simulated conditions ($Tr = 1.10$; $Pr = 3.00$). (a) Water and (b) Water + Cl^{-}.

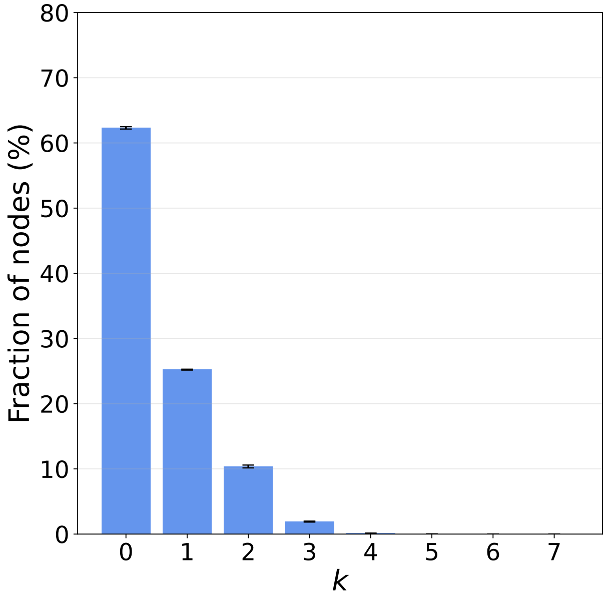

(a)

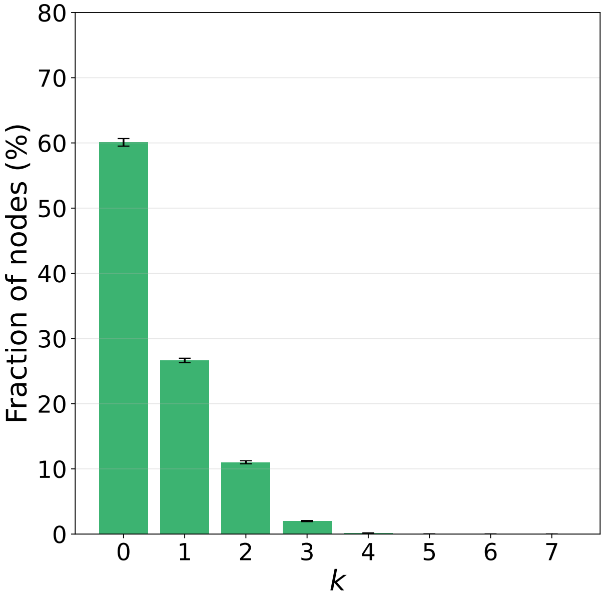

(b)

Figure S11: Intermolecular connectivity distribution under simulated conditions ($Tr = 1.10$; $Pr = 3.25$). (a) Water and (b) Water + Cl^{-}.

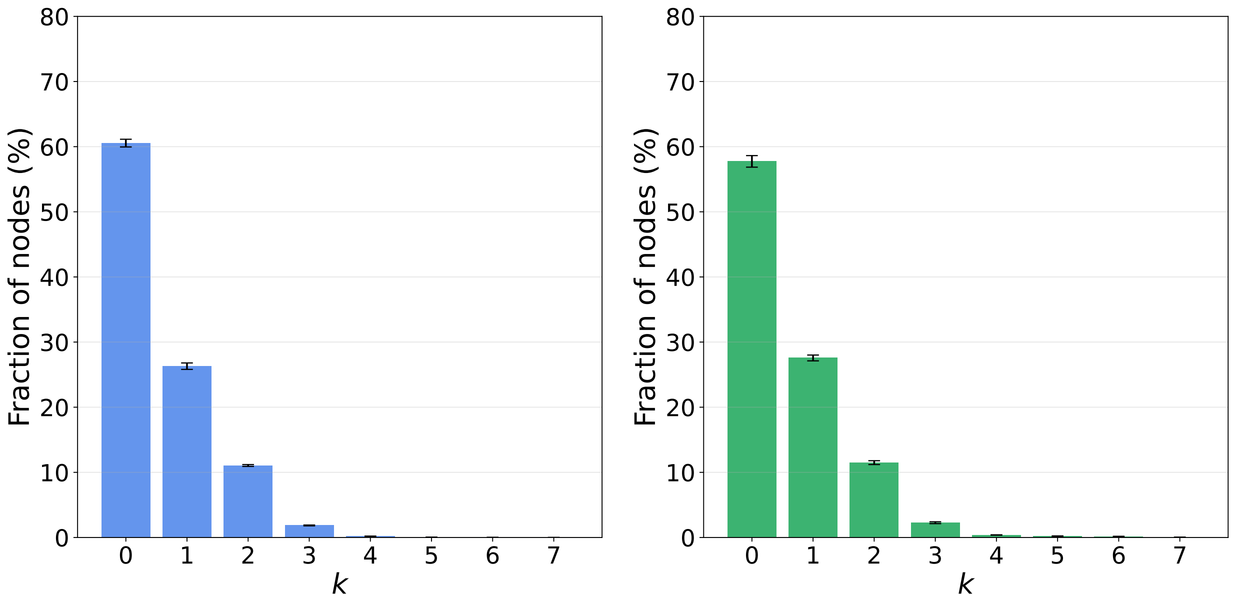

(a) (b)

Figure S12: Intermolecular connectivity distribution under simulated conditions ($Tr = 1.10$; $Pr = 3.50$). (a) Water and (b) Water + Cl^{-}.

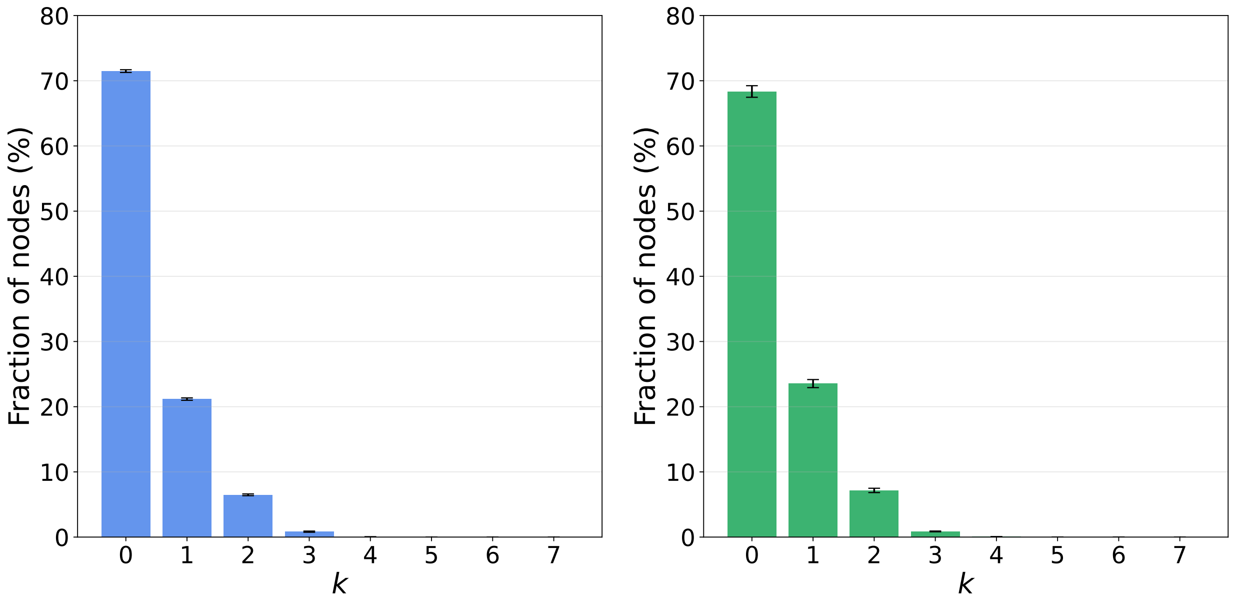

(a) (b)

Figure S13: Intermolecular connectivity distribution under simulated conditions ($Tr = 1.15$; $Pr = 3.00$). (a) Water and (b) Water + Cl^{-}.

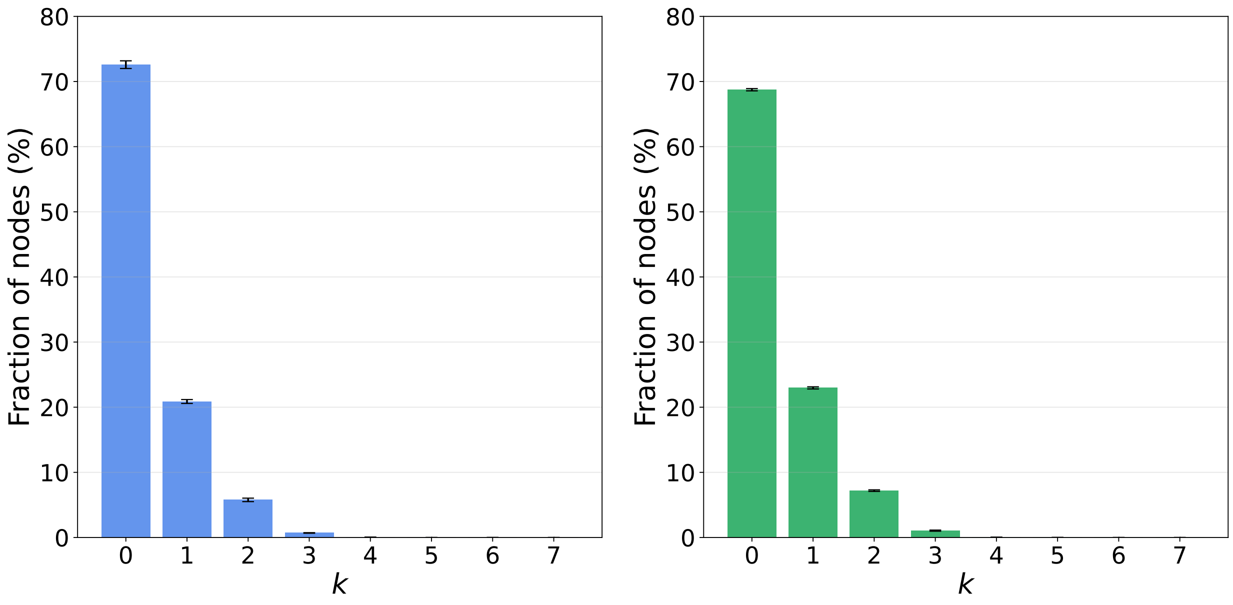

(a)

(b)

Figure S14: Intermolecular connectivity distribution under simulated conditions ($Tr = 1.15$; $Pr = 3.25$). (a) Water and (b) Water + Cl^{-}.

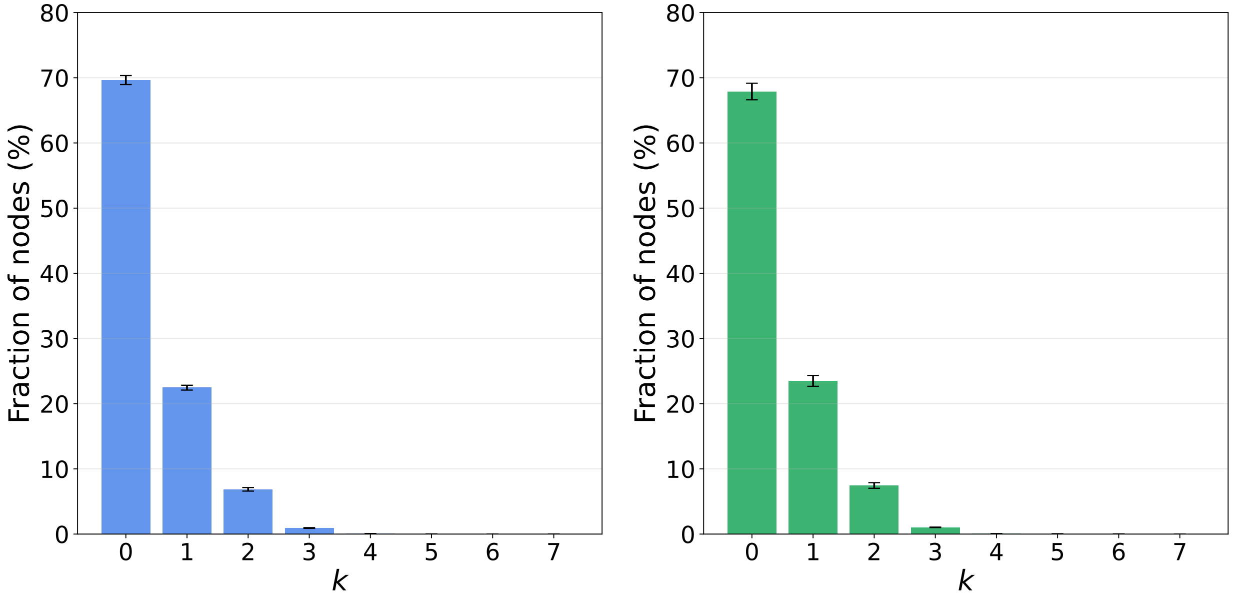

(a)

(b)

Figure S15: Intermolecular connectivity distribution under simulated conditions ($Tr = 1.15$; $Pr = 3.50$). (a) Water and (b) Water + Cl^{-}.

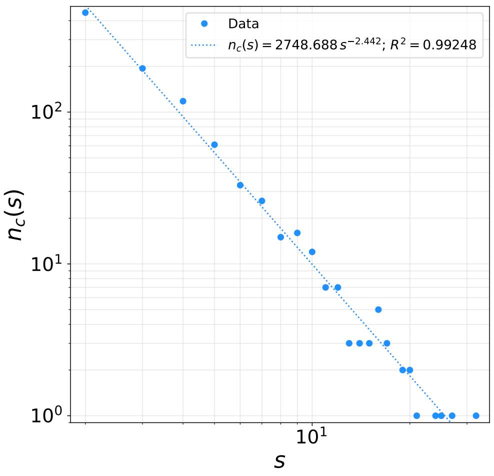

(a)

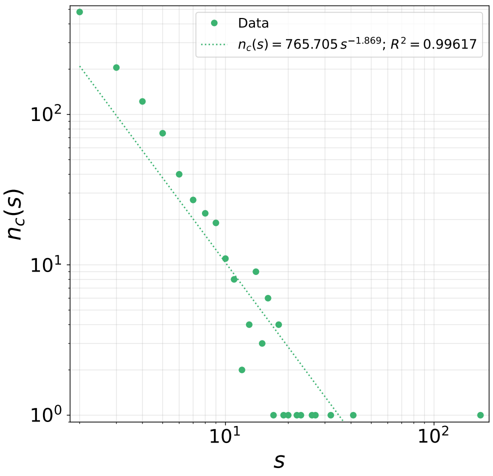

(b)

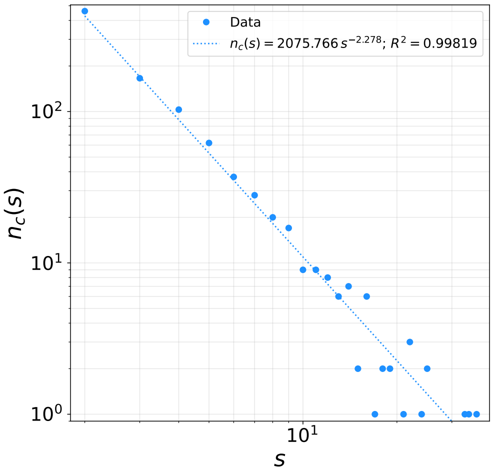

(c)

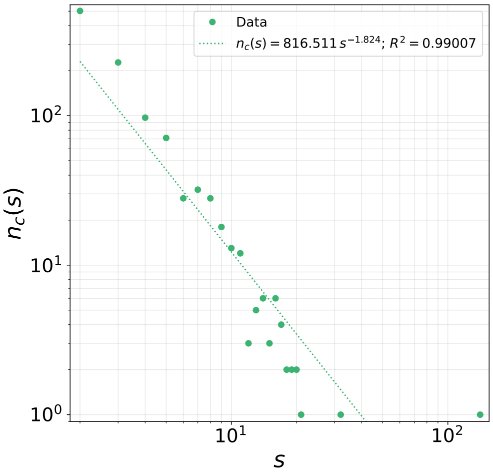

(d)

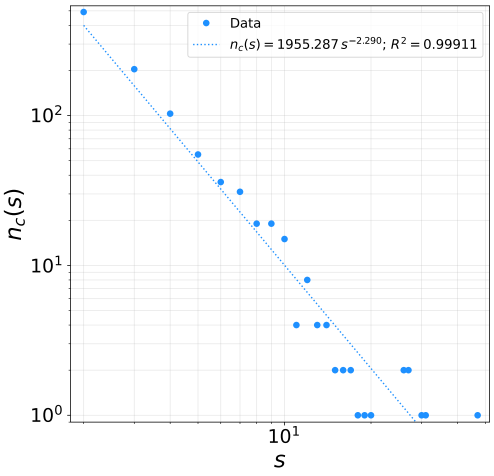

(e)

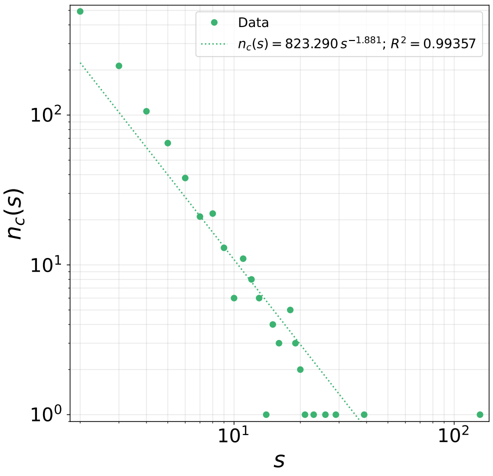

(f)

Figure S16: Connected components distribution of water under supercritical condition ($Tr = 1.05$; $Pr = 2.50$). Figures (a), (c), and (e) correspond to frames of the pure Water system, whereas figures (b), (d), and (f) correspond to frames of the Water + Cl^- system.

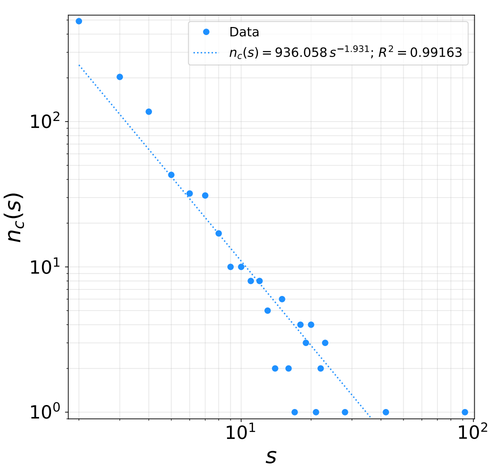

(a)

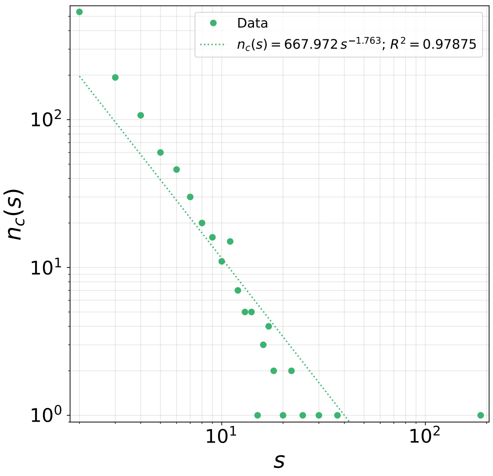

(b)

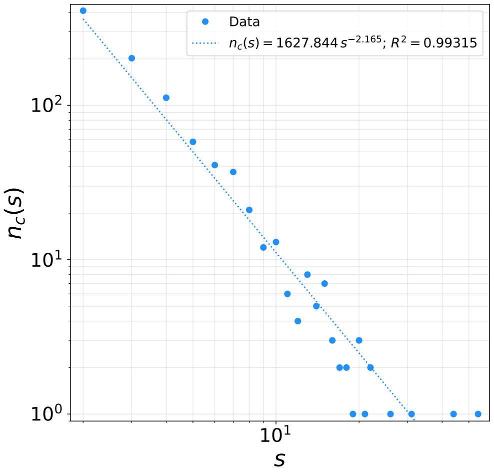

(c)

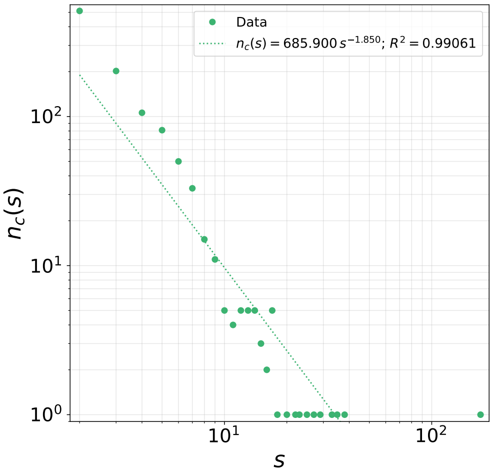

(d)

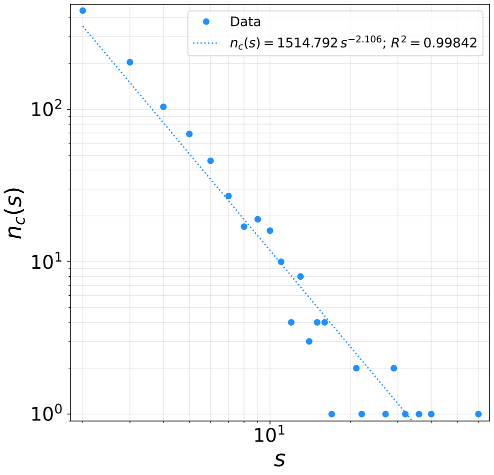

(e)

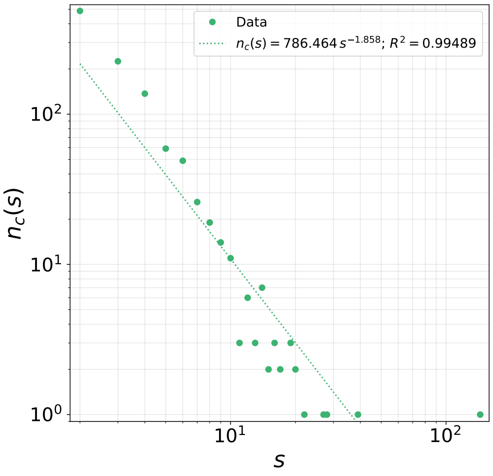

(f)

Figure S17: Connected components distribution of water under supercritical condition ($Tr = 1.05$; $Pr = 2.75$). Figures (a), (c), and (e) correspond to frames of the pure Water system, whereas figures (b), (d), and (f) correspond to frames of the Water + Cl^- system.

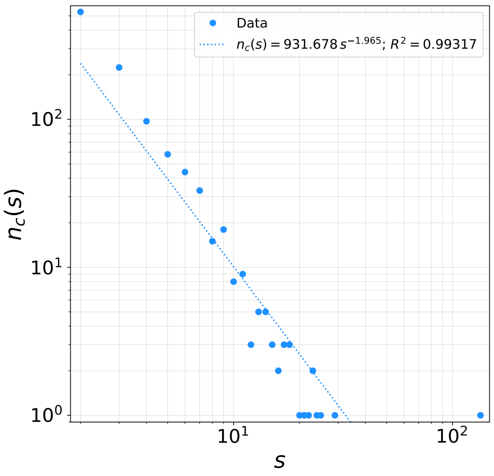

(a)

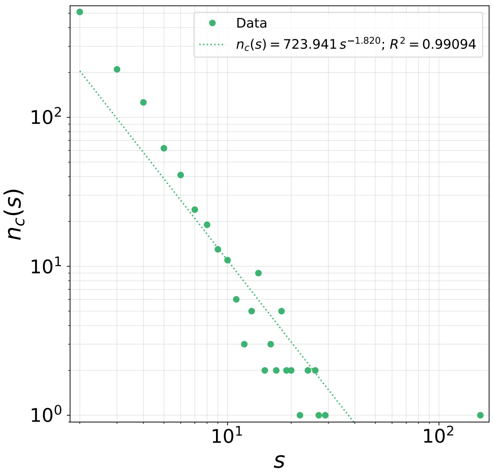

(b)

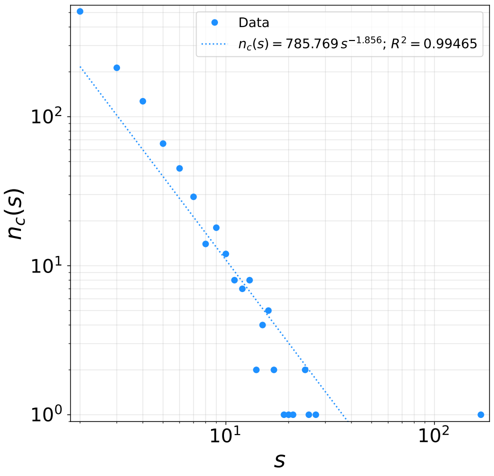

(c)

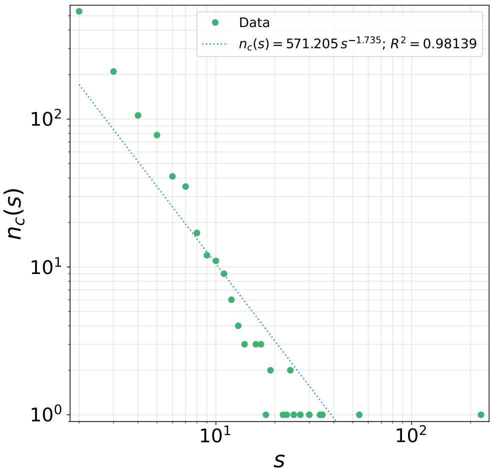

(d)

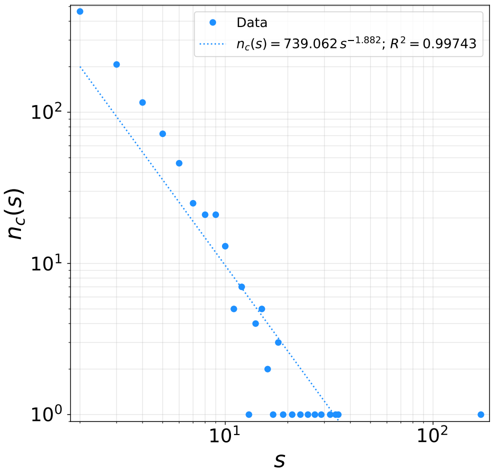

(e)

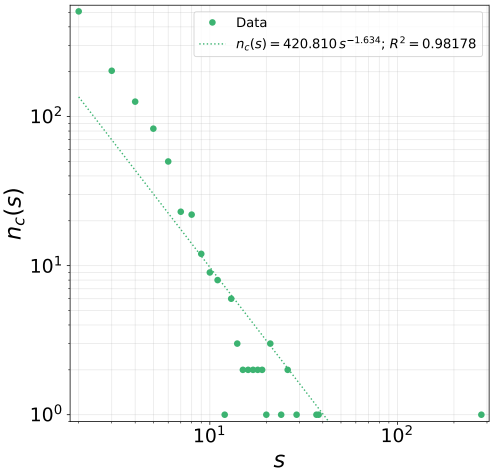

(f)

Figure S18: Connected components distribution of water under supercritical condition ($Tr = 1.05$; $Pr = 3.00$). Figures (a), (c), and (e) correspond to frames of the pure Water system, whereas figures (b), (d), and (f) correspond to frames of the Water + Cl^- system.

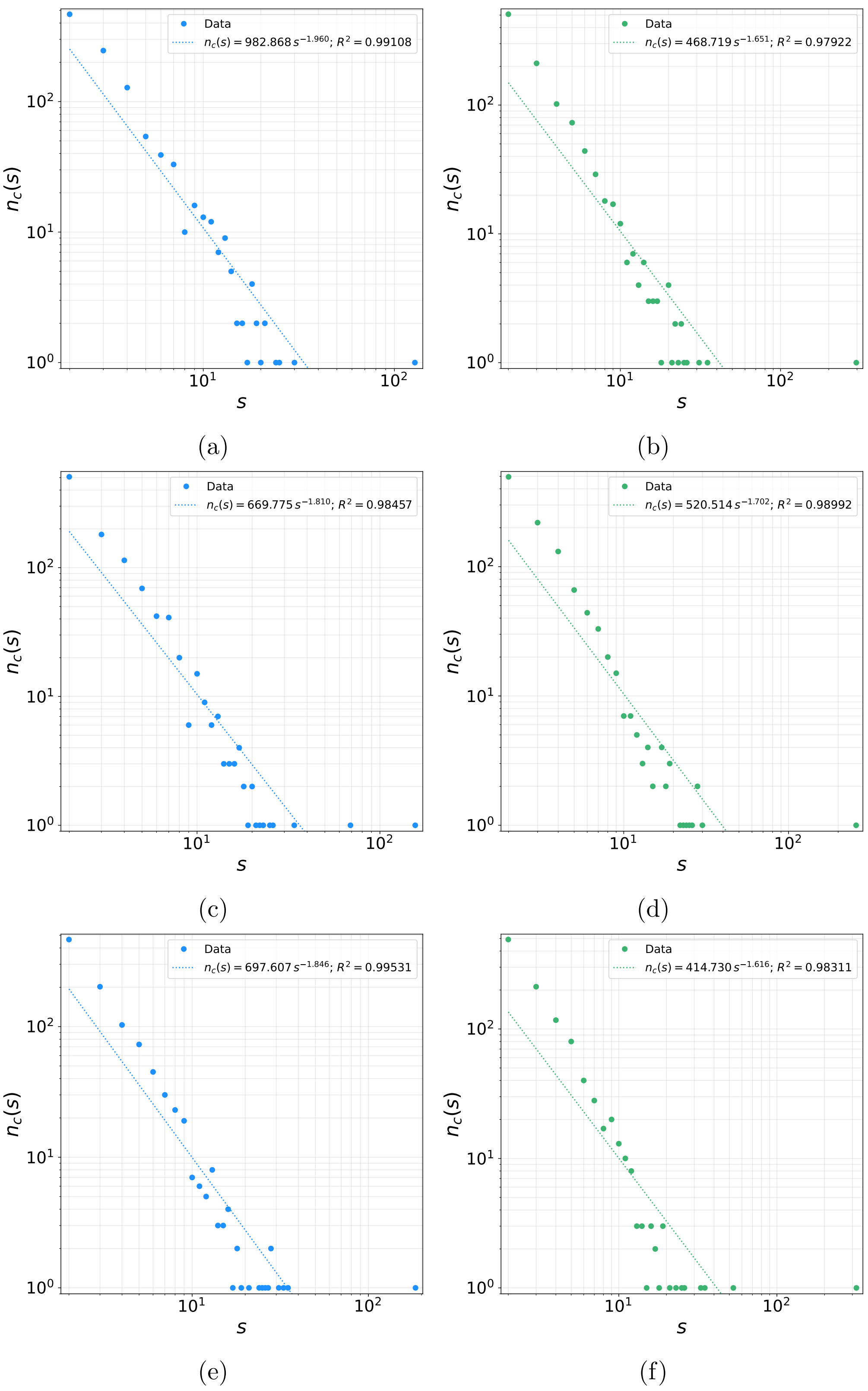

Figure S19: Connected components distribution of water under supercritical condition ($Tr = 1.05$; $Pr = 3.25$). Figures (a), (c), and (e) correspond to frames of the pure Water system, whereas figures (b), (d), and (f) correspond to frames of the Water + Cl^- system.

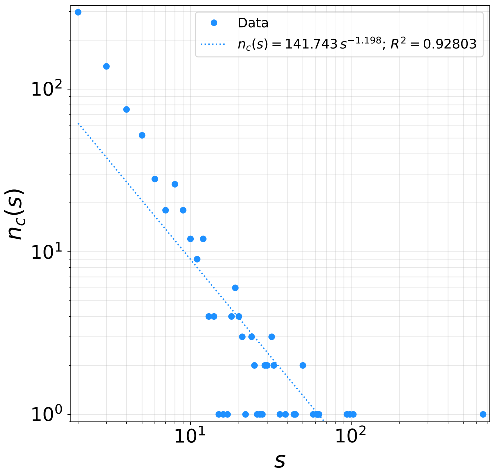

(a)

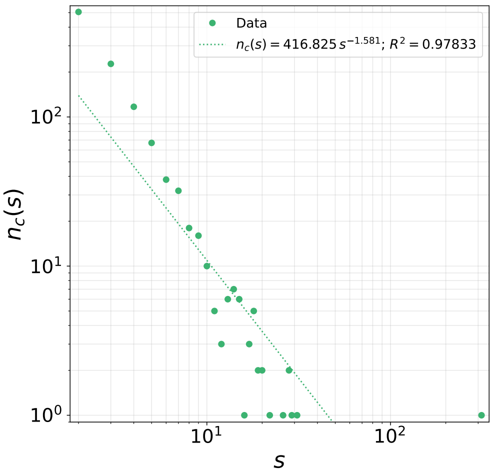

(b)

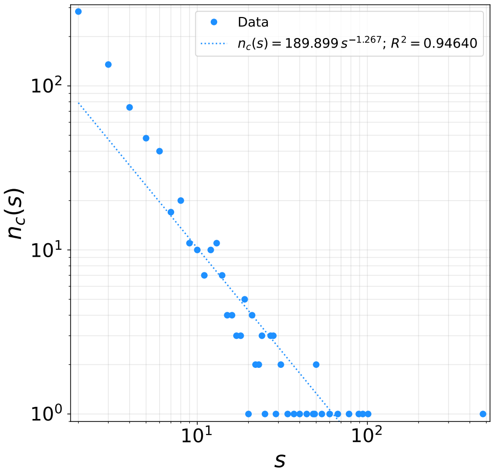

(c)

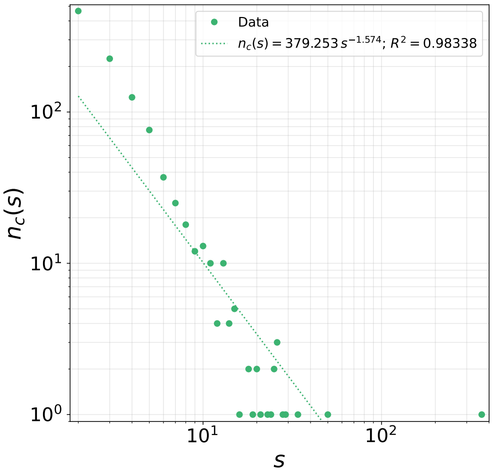

(d)

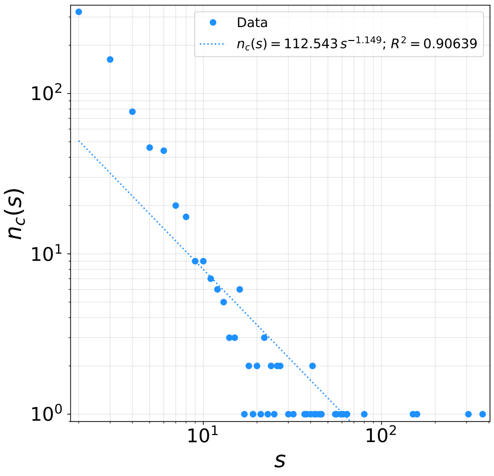

(e)

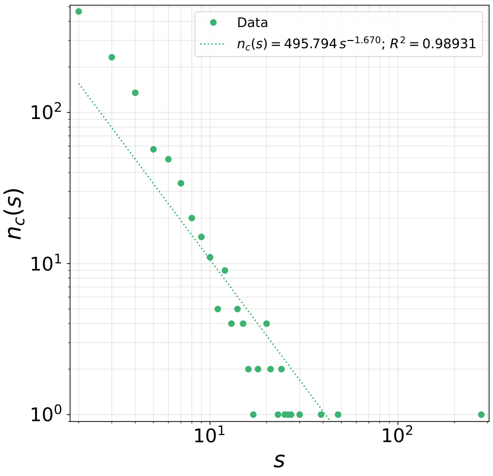

(f)

Figure S20: Connected components distribution of water under supercritical condition ($Tr = 1.05$; $Pr = 3.50$). Figures (a), (c), and (e) correspond to frames of the pure Water system, whereas figures (b), (d), and (f) correspond to frames of the Water + Cl^- system.

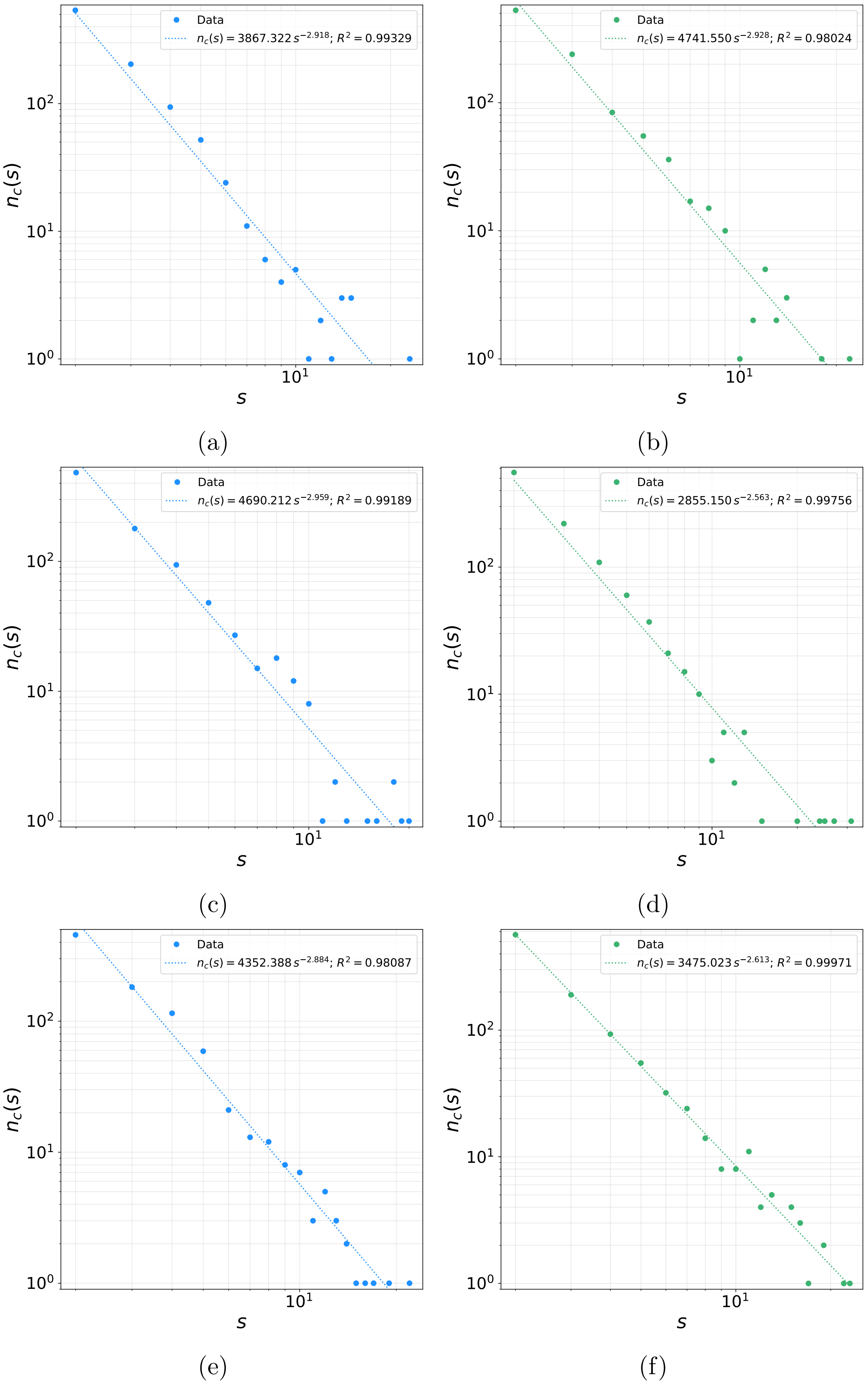

Figure S21: Connected components distribution of water under supercritical condition ($Tr = 1.10$; $Pr = 2.50$). Figures (a), (c), and (e) correspond to frames of the pure Water system, whereas figures (b), (d), and (f) correspond to frames of the Water + Cl^- system.

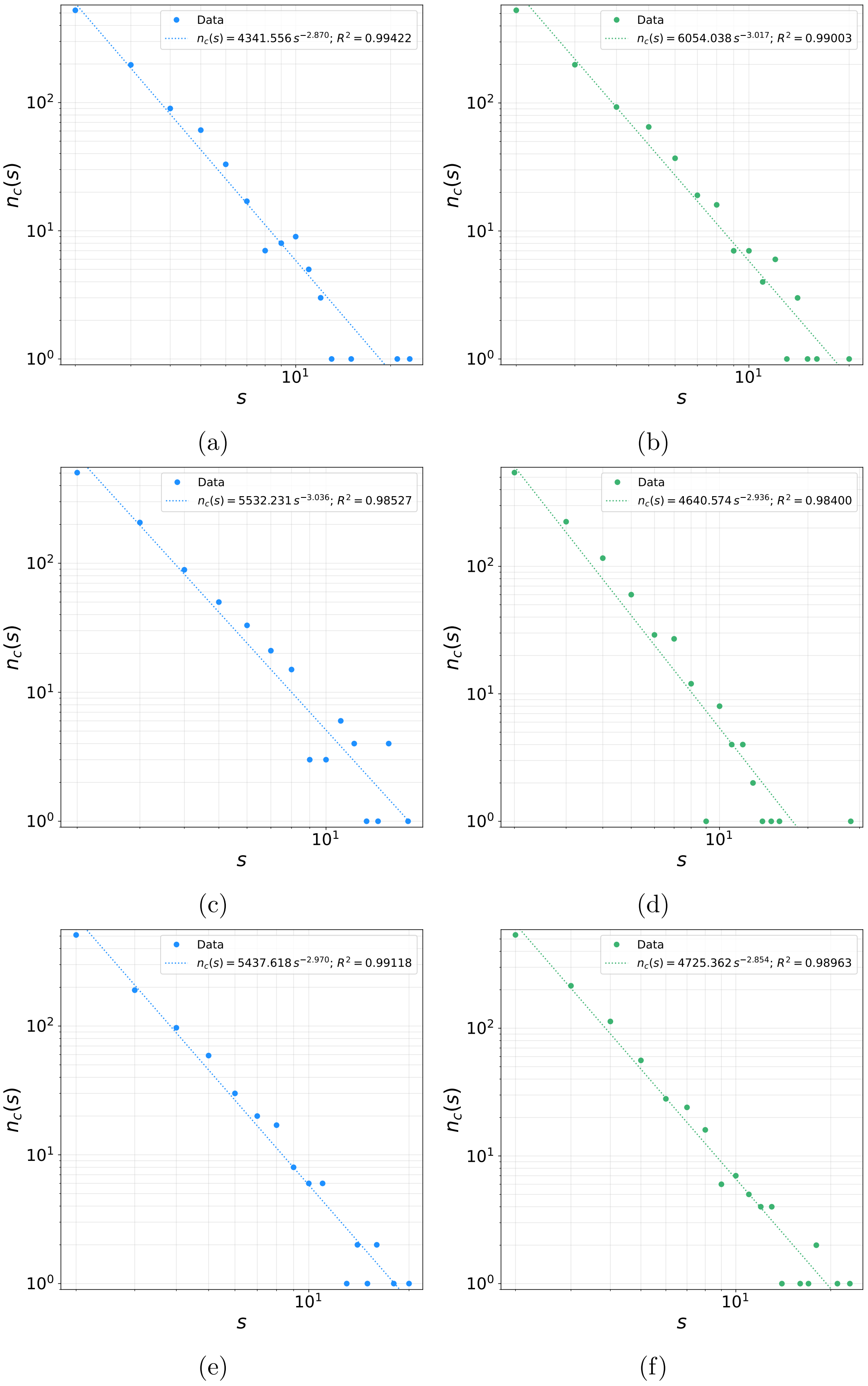

Figure S22: Connected components distribution of water under supercritical condition ($Tr = 1.10$; $Pr = 2.75$). Figures (a), (c), and (e) correspond to frames of the pure Water system, whereas figures (b), (d), and (f) correspond to frames of the Water + Cl^- system.

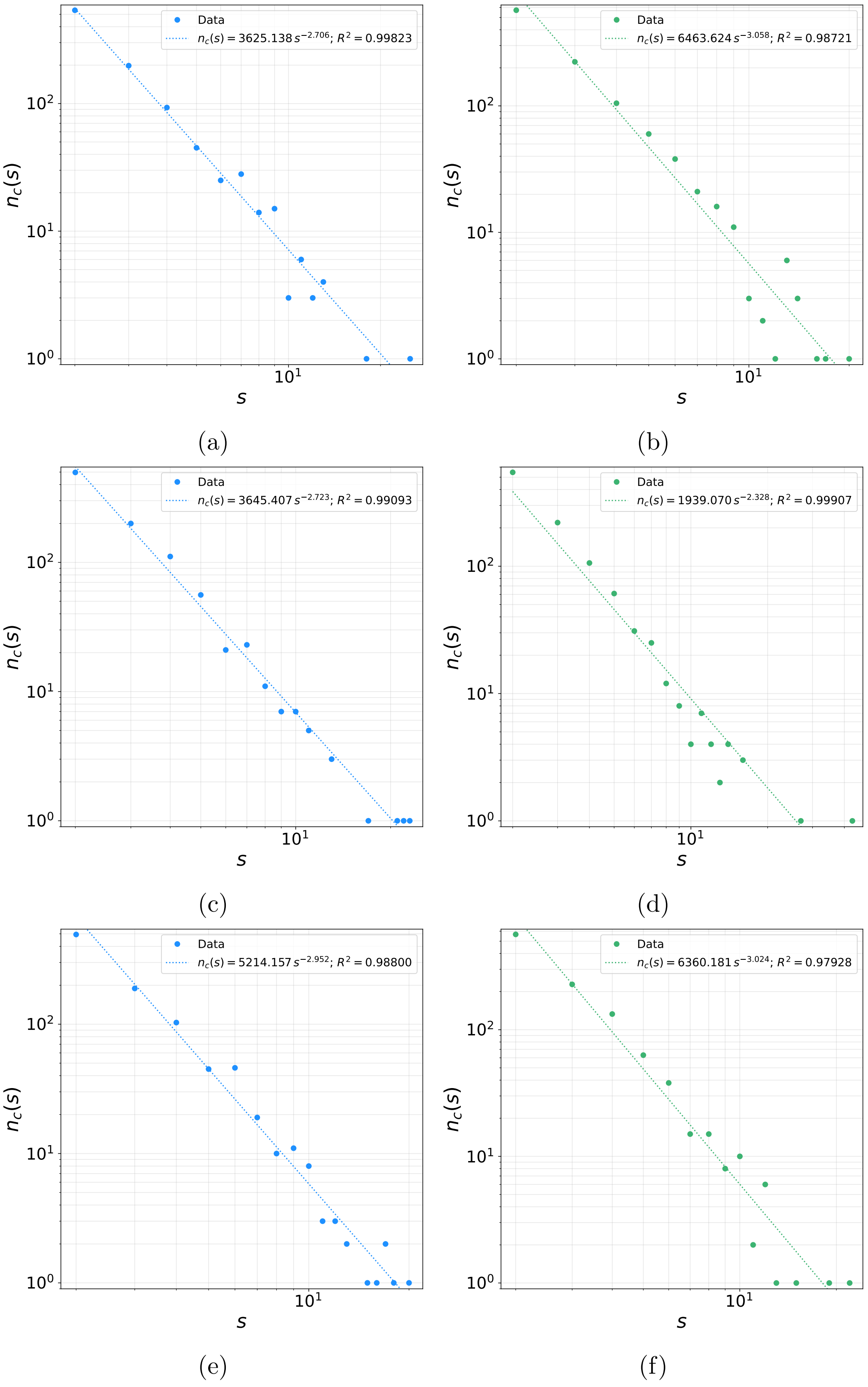

Figure S23: Connected components distribution of water under supercritical condition ($Tr = 1.10$; $Pr = 3.00$). Figures (a), (c), and (e) correspond to frames of the pure Water system, whereas figures (b), (d), and (f) correspond to frames of the Water + Cl^- system.

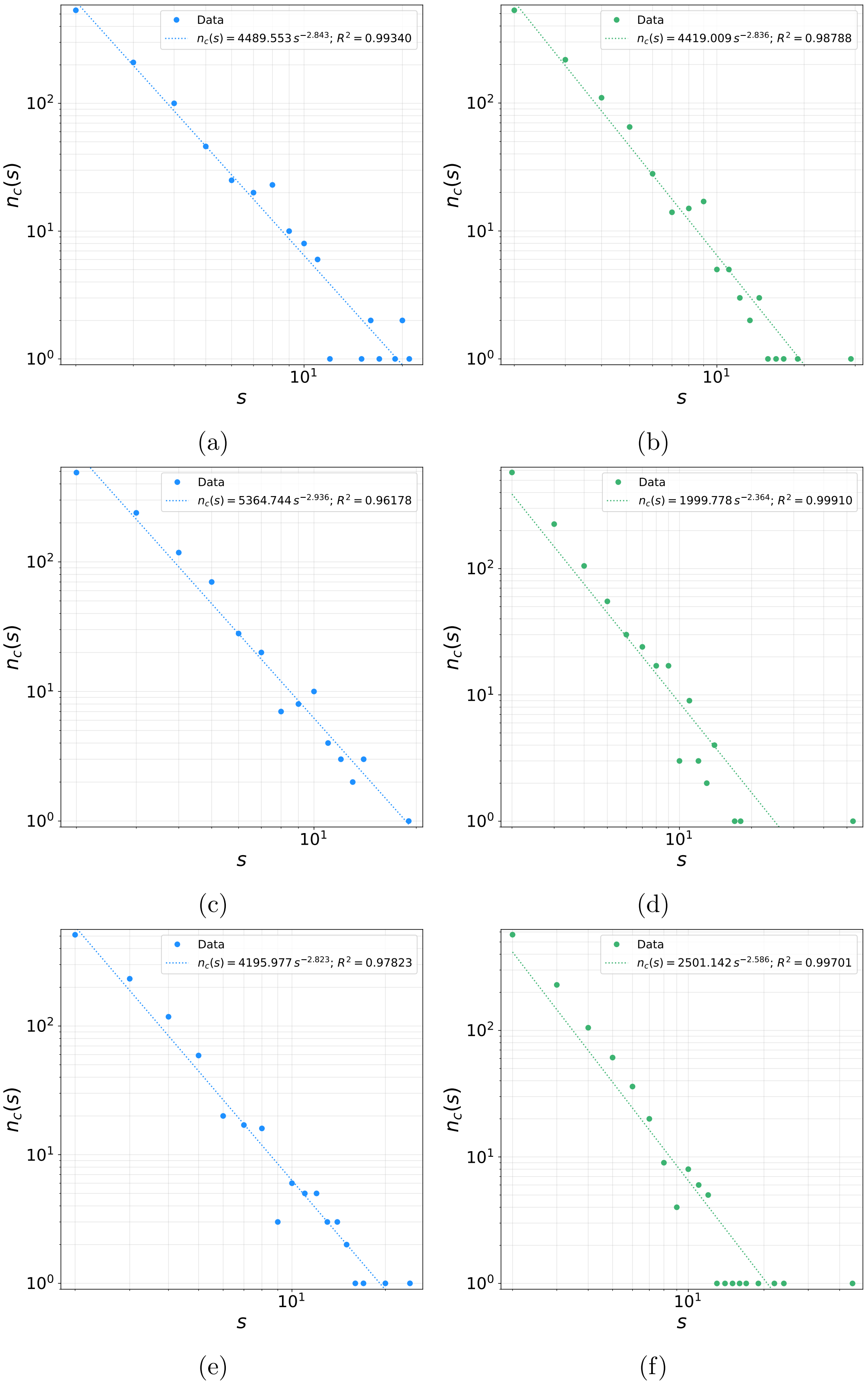

Figure S24: Connected components distribution of water under supercritical condition ($Tr = 1.10$; $Pr = 3.25$). Figures (a), (c), and (e) correspond to frames of the pure Water system, whereas figures (b), (d), and (f) correspond to frames of the Water + Cl^- system.

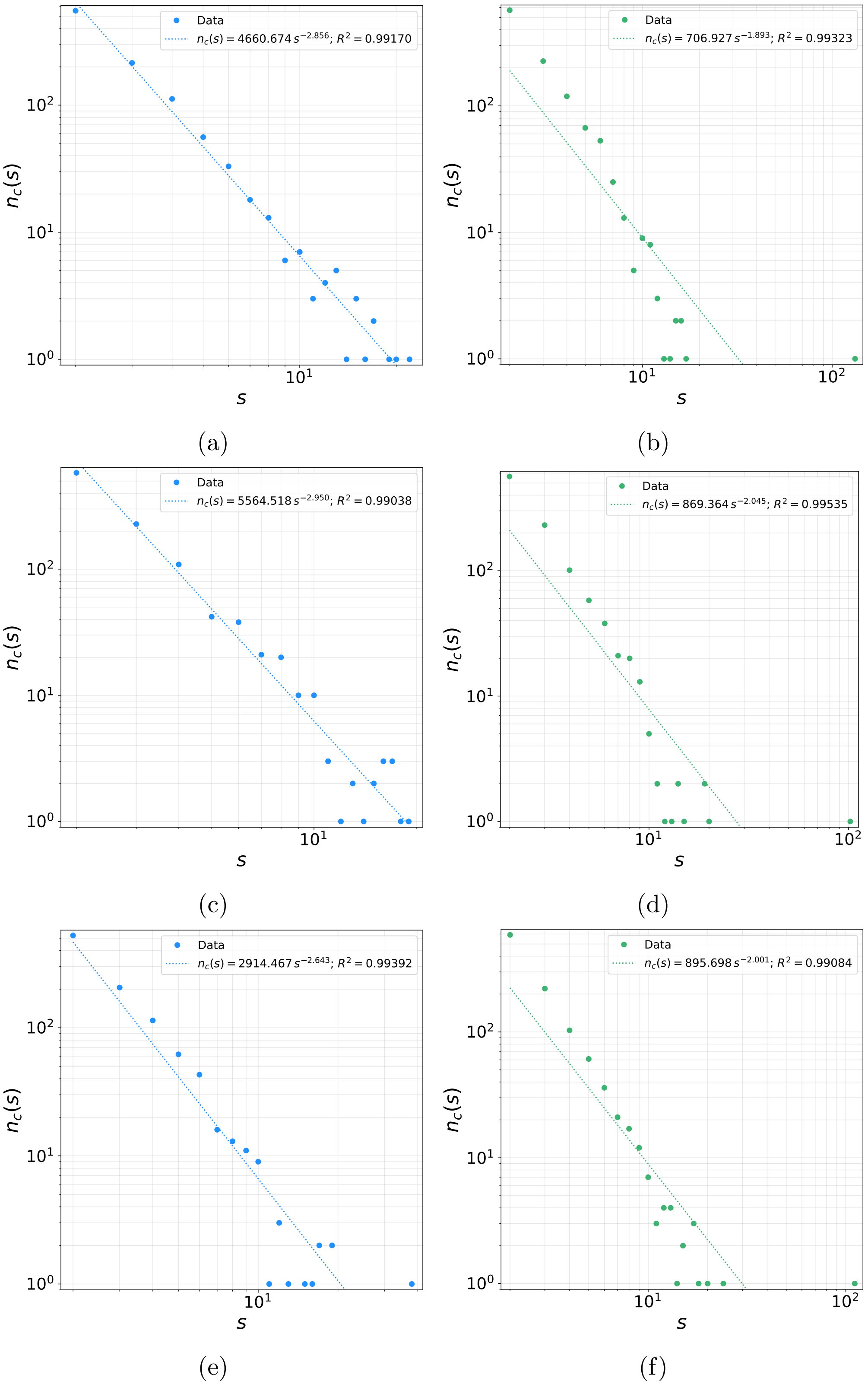

Figure S25: Connected components distribution of water under supercritical condition ($Tr = 1.10$; $Pr = 3.50$). Figures (a), (c), and (e) correspond to frames of the pure Water system, whereas figures (b), (d), and (f) correspond to frames of the Water + Cl^- system.

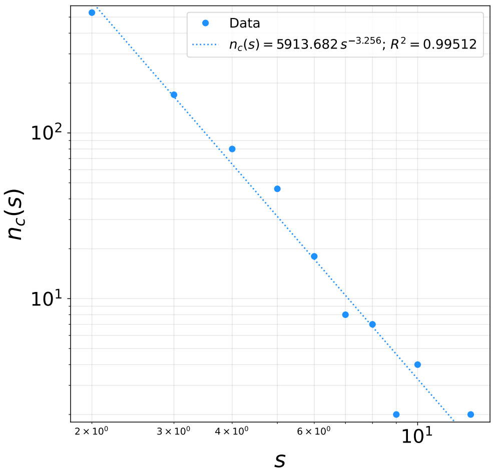

(a)

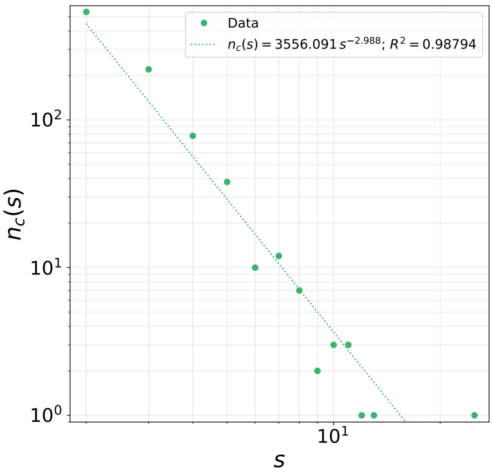

(b)

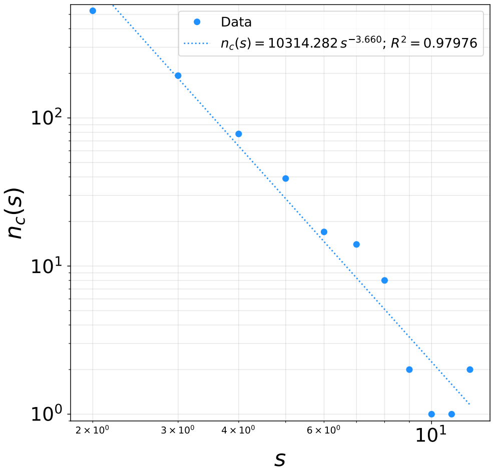

(c)

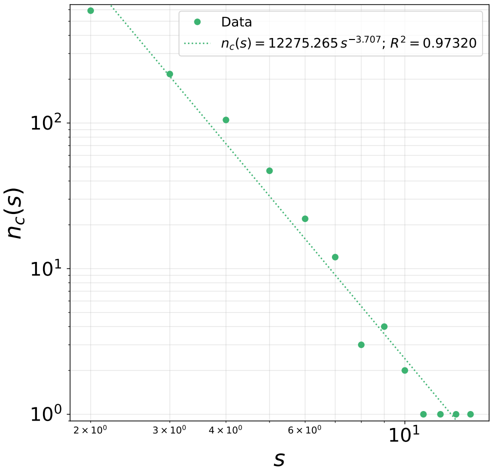

(d)

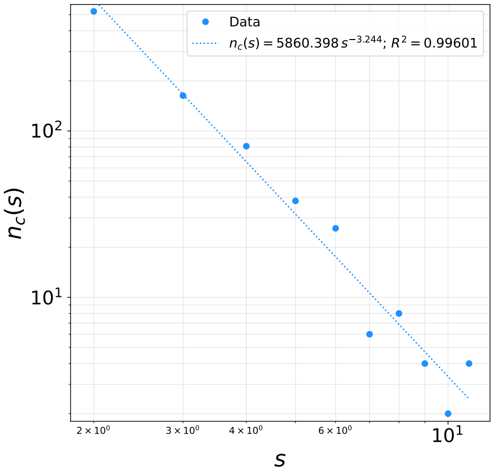

(e)

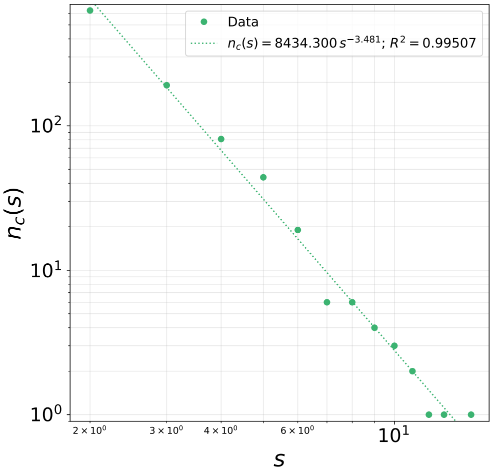

(f)

Figure S26: Connected components distribution of water under supercritical condition ($Tr = 1.15$; $Pr = 3.00$). Figures (a), (c), and (e) correspond to frames of the pure Water system, whereas figures (b), (d), and (f) correspond to frames of the Water + Cl^- system.

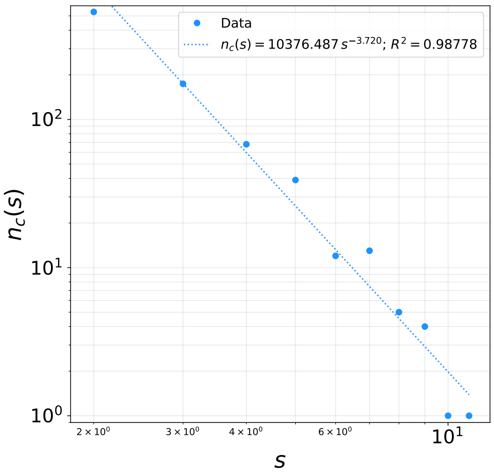

(a)

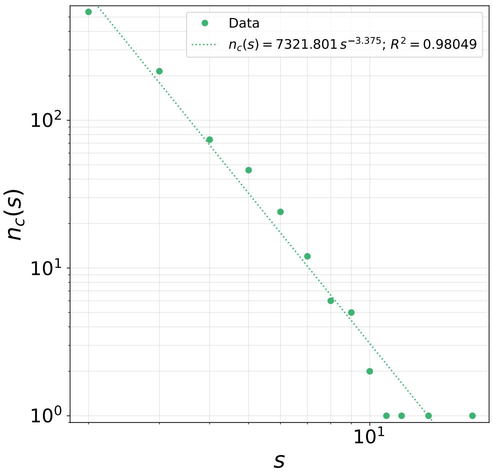

(b)

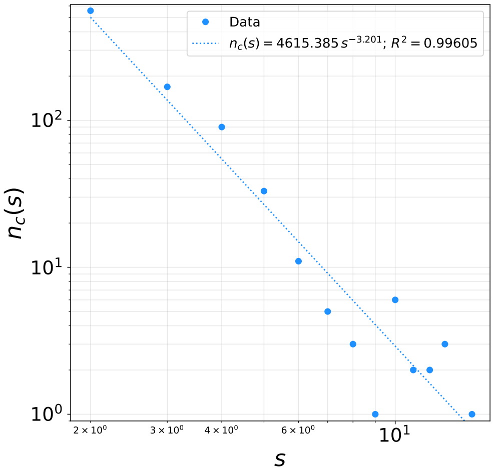

(c)

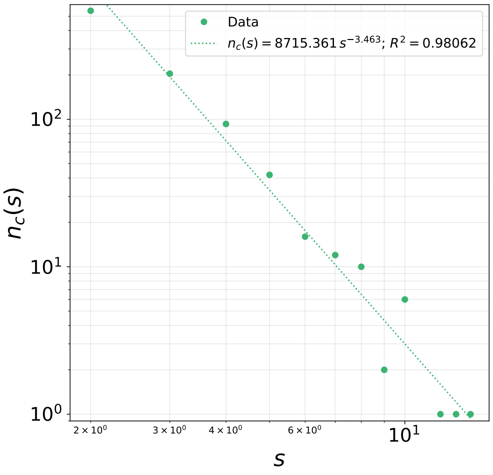

(d)

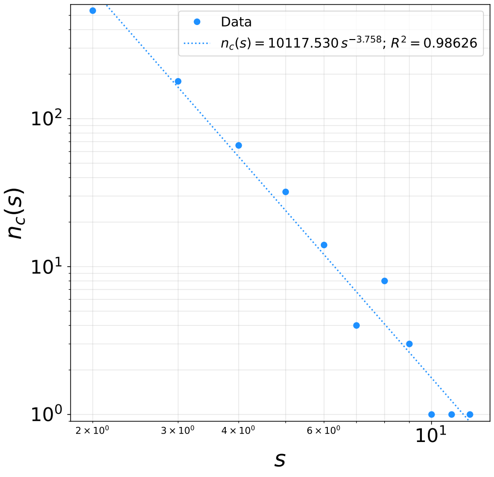

(e)

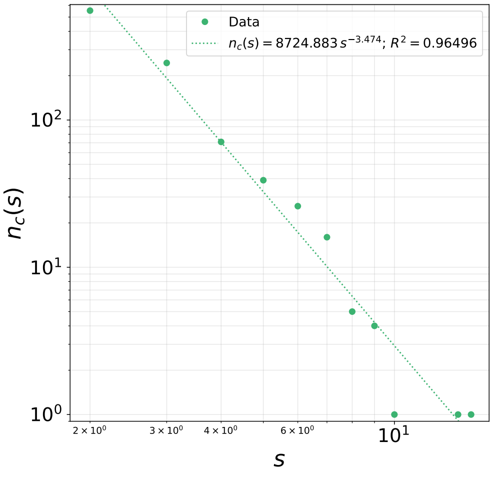

(f)

Figure S27: Connected components distribution of water under supercritical condition ($Tr = 1.15$; $Pr = 3.25$). Figures (a), (c), and (e) correspond to frames of the pure Water system, whereas figures (b), (d), and (f) correspond to frames of the Water + Cl^- system.

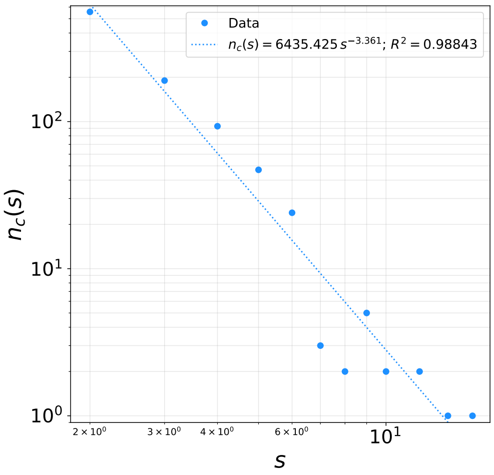

(a)

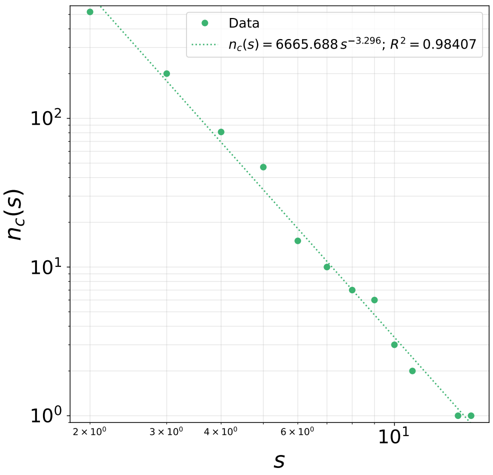

(b)

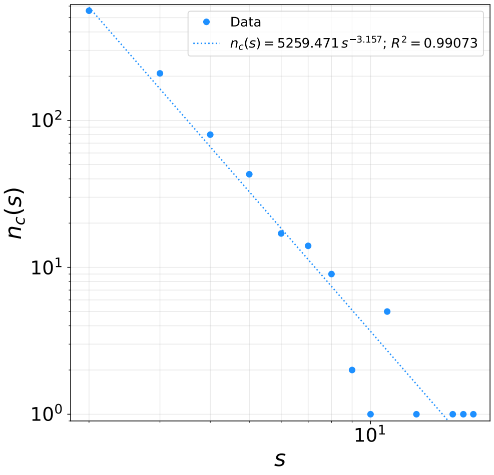

(c)

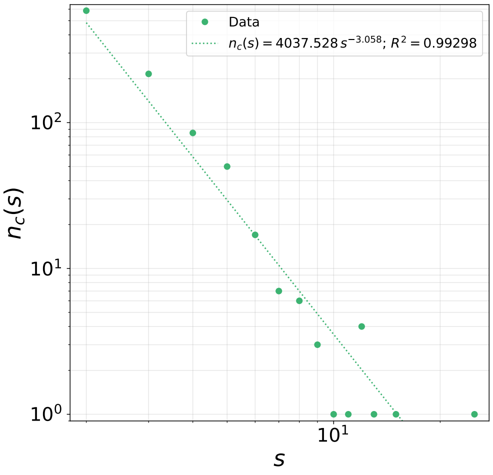

(d)

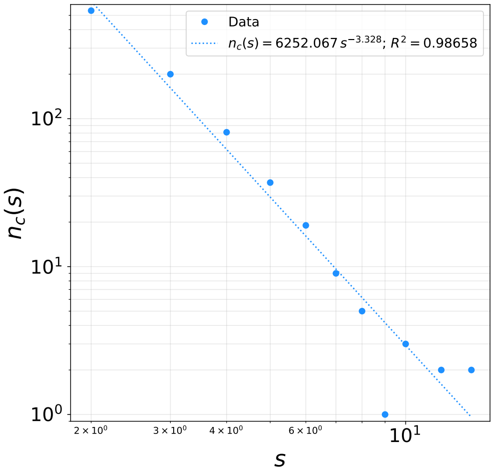

(e)

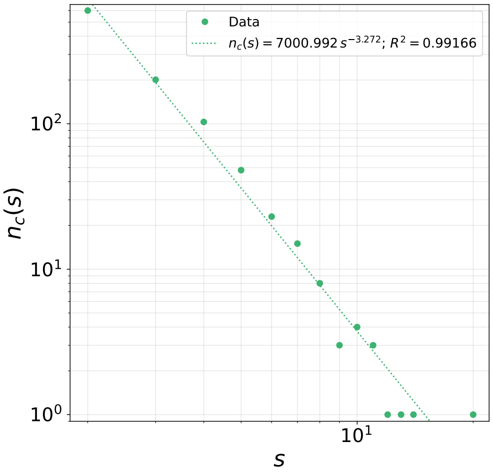

(f)

Figure S28: Connected components distribution of water under supercritical condition ($Tr = 1.15$; $Pr = 3.50$). Figures (a), (c), and (e) correspond to frames of the pure Water system, whereas figures (b), (d), and (f) correspond to frames of the Water + Cl^- system.
